# Deconvolution of sarcoma methylomes reveals varying degrees of immune cell infiltrates with association to genomic aberrations

**DOI:** 10.1186/s12967-021-02858-7

**Published:** 2021-05-12

**Authors:** Malte Simon, Sadaf S. Mughal, Peter Horak, Sebastian Uhrig, Jonas Buchloh, Bogac Aybey, Albrecht Stenzinger, Hanno Glimm, Stefan Fröhling, Benedikt Brors, Charles D. Imbusch

**Affiliations:** 1 Division of Applied Bioinformatics, German Cancer Research Center (DKFZ), Heidelberg, Germany; 2 Division of Translational Medical Oncology, National Center for Tumor Diseases, German Cancer Research Center, Heidelberg, Germany; 3 Faculty of Biosciences, Heidelberg University, Heidelberg, Germany; 4 Institute of Pathology, University Hospital Heidelberg, Heidelberg, Germany; 5 German Cancer Consortium (DKTK), German Cancer Research Center (DKFZ), Heidelberg, Germany; 6 Department of Translational Medical Oncology, NCT Dresden, Dresden, Germany; 7 University Hospital Carl Gustav Carus, Technical University Dresden, Dresden, Germany

**Keywords:** Sarcoma, Deconvolution, Survival analysis, Tumor-infiltrating leukocytes

## Abstract

**Background:**

Soft-tissue sarcomas (STS) are a heterogeneous group of mesenchymal tumors for which response to immunotherapies is not well established. Therefore, it is important to risk-stratify and identify STS patients who will most likely benefit from these treatments.

**Results:**

To reveal shared and distinct methylation signatures present in STS, we performed unsupervised deconvolution of DNA methylation data from the TCGA sarcoma and an independent validation cohort. We showed that leiomyosarcoma can be subclassified into three distinct methylation groups. More importantly, we identified a component associated with tumor-infiltrating leukocytes, which suggests varying degrees of immune cell infiltration in STS subtypes and an association with prognosis. We further investigated the genomic alterations that may influence tumor infiltration by leukocytes including *RB1* loss in undifferentiated pleomorphic sarcomas and *ELK3* amplification in dedifferentiated liposarcomas.

**Conclusions:**

In summary, we have leveraged unsupervised methylation-based deconvolution to characterize the immune compartment and molecularly stratify subtypes in STS, which may benefit precision medicine in the future.

**Supplementary Information:**

The online version contains supplementary material available at 10.1186/s12967-021-02858-7.

## Background

Soft-tissue sarcomas (STS) are rare cancers of mesenchymal origin that represent < 1% of adult solid malignancies. Their high diversity in terms of genetic aberrations and histological appearance results in a subclassification into more than 70 subtypes [1]. Recently, the TCGA consortium released a study comprising the characterization of 206 sarcomas from six subtypes including dedifferentiated liposarcoma (DDLPS), leiomyosarcoma (LMS), undifferentiated pleomorphic sarcoma (UPS), myxofibrosarcoma (MFS), malignant peripheral nerve sheath tumor (MPNST) and synovial sarcoma (SS). By analyzing genetic, epigenetic, mRNA and protein expression data, the authors stated that subtypes with complex karyotypes are mostly driven by copy number alterations instead of mutations, and that the presence of certain inferred immune cell types and methylation states associates with disease-specific survival [2]. Several clinical trials on immunotherapies in STS have found low overall response rates, which highlights the importance of a more detailed characterization of immune infiltrates and the development of robust predictors of clinical benefit in these tumors [3, 4]. While a global comparison of immunogenicity in different tumor types has been previously presented, to date, no study focused on sarcomas [5, 6].

In this study, we reanalyzed the TCGA-SARC dataset from an epigenetic perspective by employing unsupervised deconvolution of the methylation data to discover shared as well as subtype-specific methylation profiles. By correlating distinct methylation changes with mRNA abundances, we derived gene signatures for each profile and showed their biological relevance and usability for subclassification. Importantly, we identified an immune cell-associated component that implies varying degrees of immune cell infiltration in STS with enrichment in UPS, DDLPS and MFS cases, whereas it was substantially lower in LMS and SS. In addition genomic aberrations could be identified that harbour the potential to influence tumor infiltration. We validated the immune-cell associated signature as well as associated genomic aberrations in independent cohorts.

## Results

### Deconvolution of methylation data results in subtype-specific patterns

**Fig. 1:**
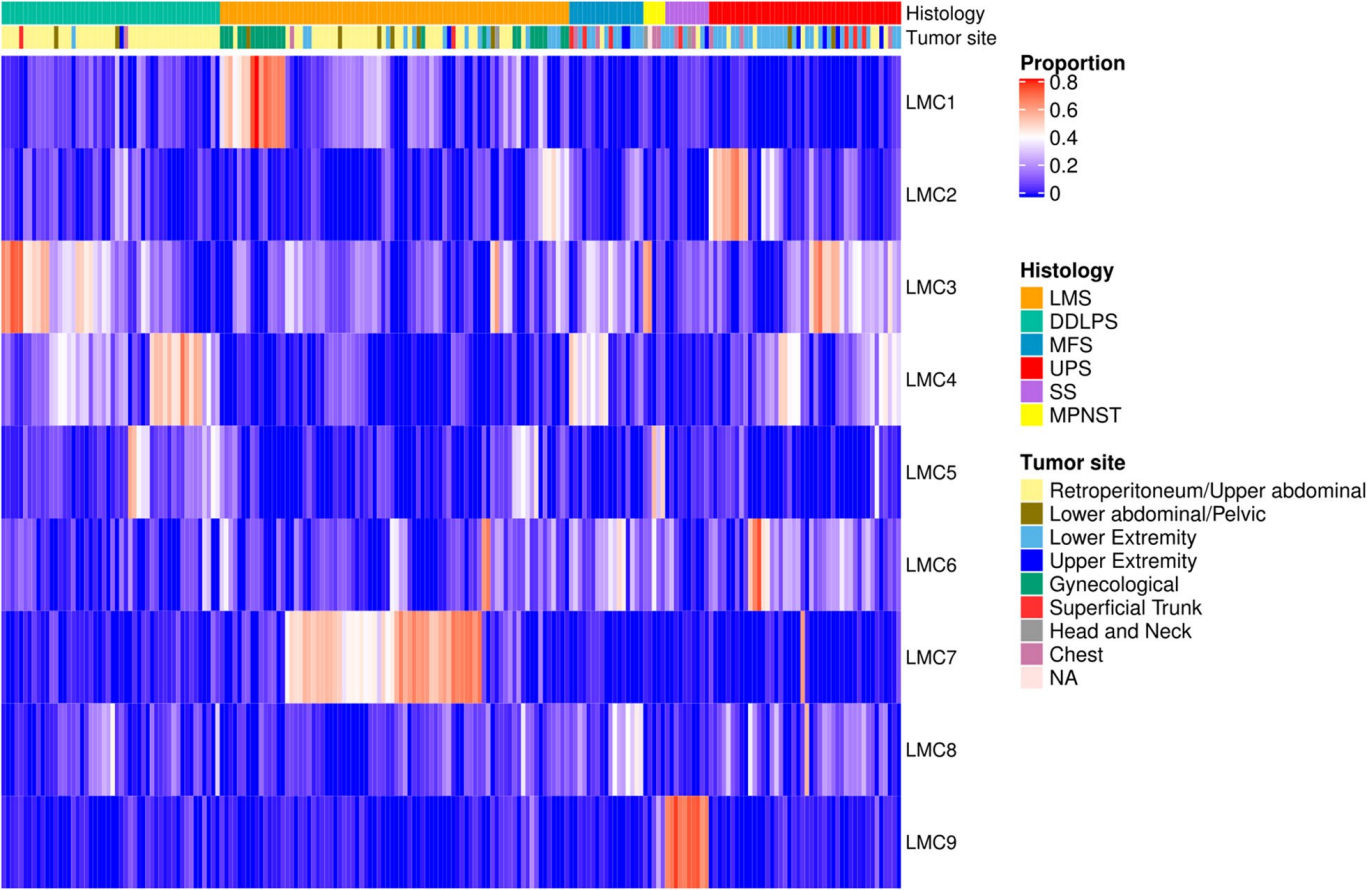
MeDeCom deconvolves subtype-specific methylation patterns. Proportions for the deconvolution of TCGA-SARC methylation data using 9 LMCs and λ = 0.01 are shown. Unsupervised deconvolution based on non-negative matrix factorization resulted in methylation components associated with histological subtypes and tumor tissue sites. LMS, leiomyosarcoma; DDLPS, dedifferentiated liposarcoma; MFS, myxofibrosarcoma; UPS, undifferentiated pleomorphic sarcoma; SS, synovial sarcoma; MPNST, malignant peripheral nerve sheath tumor

To identify shared methylation patterns (hereafter referred to as latent methylation components, LMCs), we analyzed the TCGA sarcoma methylation data aggregated within equidistant and non-overlapping genomic windows and performed a deconvolution using MeDeCom [7]. We chose a factorization into nine LMCs based on a low cross-validation error and high stability of the resulting methylation patterns (Additional file 1: Figure S1). Hierarchical clustering on the proportions of the LMCs, which represent the relative occurrence of the respective patterns in the tumor samples, showed clear associations to histopathological subtypes for several components (Fig. 1). The strongest association was found for LMC9 and synovial sarcoma (point biserial correlation coefficient (*r*_*pb*_) = 0.97) reflecting the dramatic changes to their methylome, which occur in this subtype as a consequence of an SS18-SSX gene fusion [8]. We further observed a global hypomethylation in SS compared to the other subtypes (Additional file 1: Figure S2). LMC1 was associated with uterine LMS (ULMS, *r*_*pb*_ = 0.72), whereas LMC7 represented a methylation pattern common in most nongynecological LMS (STLMS, *r*_*pb*_ = 0.81). Although having a weaker association, LMC2 was predominantly shared among UPS cases (*r*_*pb*_ = 0.36), and LMC4 showed the strongest association with DDLPS (*r*_*pb*_ = 0.46). Whereas, LMCs 3, 5, 6, and 8 were shared among the different sarcoma subtypes.

We validated our findings using an independent methylation dataset (MASTER) from 56 sarcoma samples (Additional file 1: Figure S3). In concordance with the deconvolution results from TCGA-SARC, subtype-associated methylation patterns were consistently observed for LMS and SS. In addition, we also found unique methylation patterns for gastrointestinal stromal tumors (GIST), solitary fibrous tumors (SFT) and myxoid liposarcoma (MLS). Hierarchical clustering of the LMCs from the independent deconvolution of TCGA-SARC and HIPO showed a high similarity of the methylation patterns associated with the subtypes present in both datasets (LMS and SS, Additional file 1: Figure S4). This confirms that these subtypes have distinct methylation changes, which are consistently observed across datasets.

### Integration of methylation and gene expression data defines three molecular LMS subgroups

LMS cases were assigned into three groups based on their LMC proportions: LMS group 1 (STLMS-associated) was defined by samples with a LMC7 proportion greater than LMC1, LMS group 2 (ULMS-associated) as LMC1 proportion greater than LMC7, and samples with a proportion smaller than 0.2 in both were defined as LMS group 3. To further characterize these subgroups, we applied a workflow to extract the LMC-specific methylation and integration of the mRNA expression (Additional file 1: Figure S5).

For each LMC, we extracted genomic windows with a methylation difference below − 0.2 (hypomethylated) or above 0.2 (hypermethylated) in one LMC compared to all other LMCs. This resulted in a mean of ~ 1000 windows per LMC, to which we subsequently refer to as variably methylated regions (VMRs) (Additional file 1: Figure S6A). Next, we searched for genomic overlaps of VMRs with known genes and calculated the correlation between methylation and gene expression. This procedure resulted in four LMC-specific combinations: hypo- or hypermethylated VMRs that are either positively or negatively correlated with gene expression, respectively (Additional file 1: Figure S6B). In particular, we were interested in methylation changes resulting in upregulation of gene expression, since these provide insights on gene activity in the LMC-associated tumors and might constitute potential new biomarkers. We calculated the Pearson’s correlation between methylation and gene expression to further filter VMRs. The cutoff for a significant correlation was greater than 0.3 for hypermethylation-upregulation or smaller than -0.3 for hypomethylation-upregulation.

**Fig. 2:**
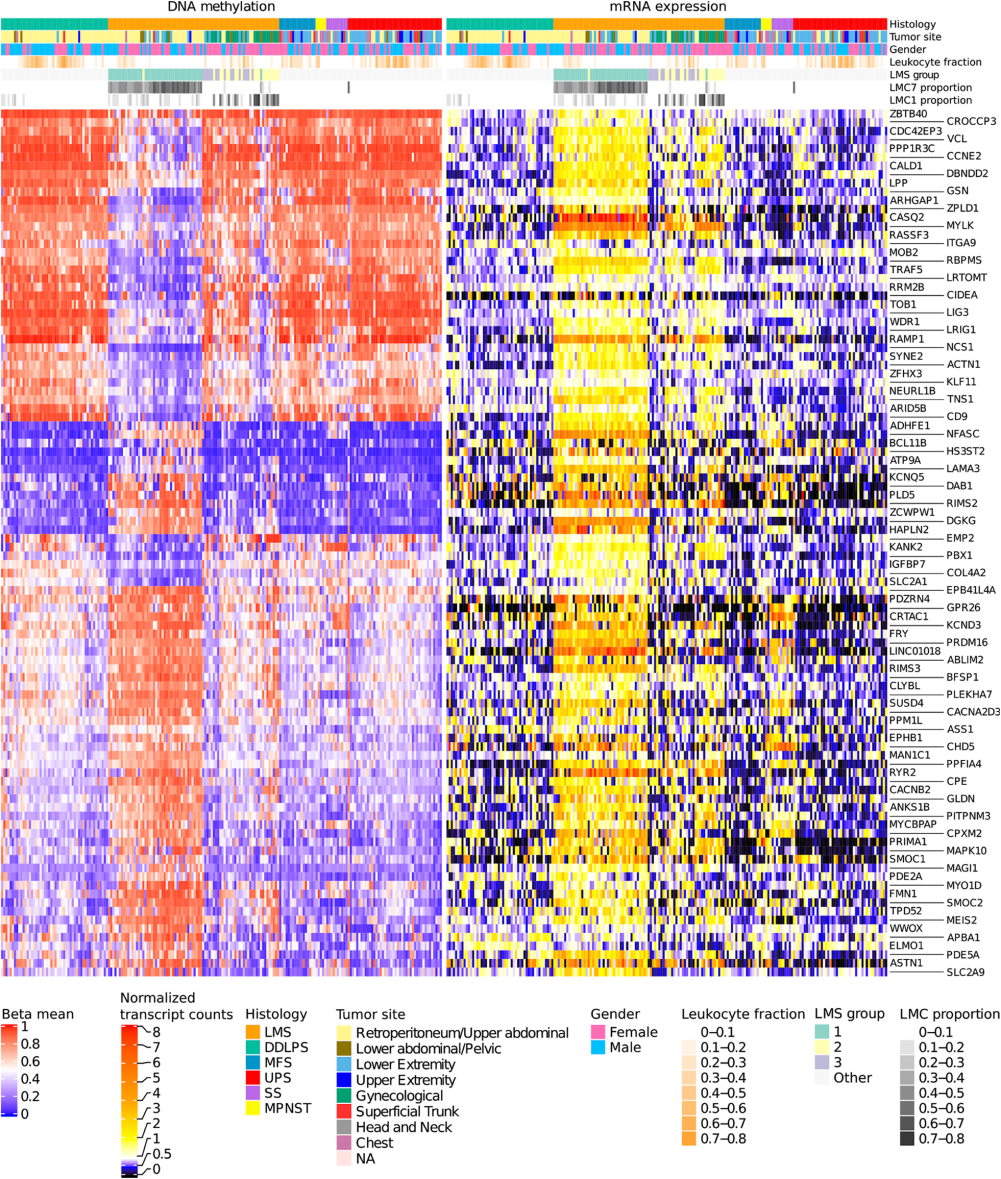
STLMS core signature. The heatmap shows DNA methylation and mRNA expression for the top 100 genes in samples with a high proportion of LMC7. Samples are clustered within the same histological subtype in columns, and genes are clustered in rows. The three LMS subgroups shown in the annotations were assigned based on the proportions of LMC1 and 7. Group 1, associated with LMC7 mainly comprised STLMS, whereas group 2 (LMC1) was enriched for tumors belonging to ULMS. Leukocyte fraction scores are shown as estimated by [2]

Following this approach, we identified 100 genes for STLMS-associated LMC7 (Fig. 2), which we termed as ‘STLMS core signature’. 42 of these genes had hypomethylated and 58 had hypermethylated VMRs. 55% of the LMS tumors had the highest proportion in LMC7, with the majority of the samples belonging to retroperitoneal/upper abdominal region and only one uterine sample (LMS group 1).

The STLMS core signature also contained the previously known immunohistochemical markers such as *MYLK* and *CASQ2* [9, 10]. Next, we performed functional annotation of these genes and found enrichments in gene sets associated with muscle function (Additional file 2: Table S1) such as focal adhesion (*P* < 0.004), actin binding (*P* < 0.007) and muscle contraction (*P* = 0.131) (Fisher's exact test and Benjamini - Hochberg correction) indicating that LMS group 1 is mostly associated with smooth muscle function.

ULMS-associated LMC1 had the highest contribution in 26% of the LMS tumors (17 ULMS and 4 STLMS, LMS group 2). For this LMC, 42 hypermethylated and 31 hypomethylated genes passed our filtering criteria (Additional file 1: Figure S7, LMC1 signature). Enrichment analysis of LMC1 genes also pointed towards muscle function and differentiation signature, although the results were not statistically significant.

**Fig. 3:**
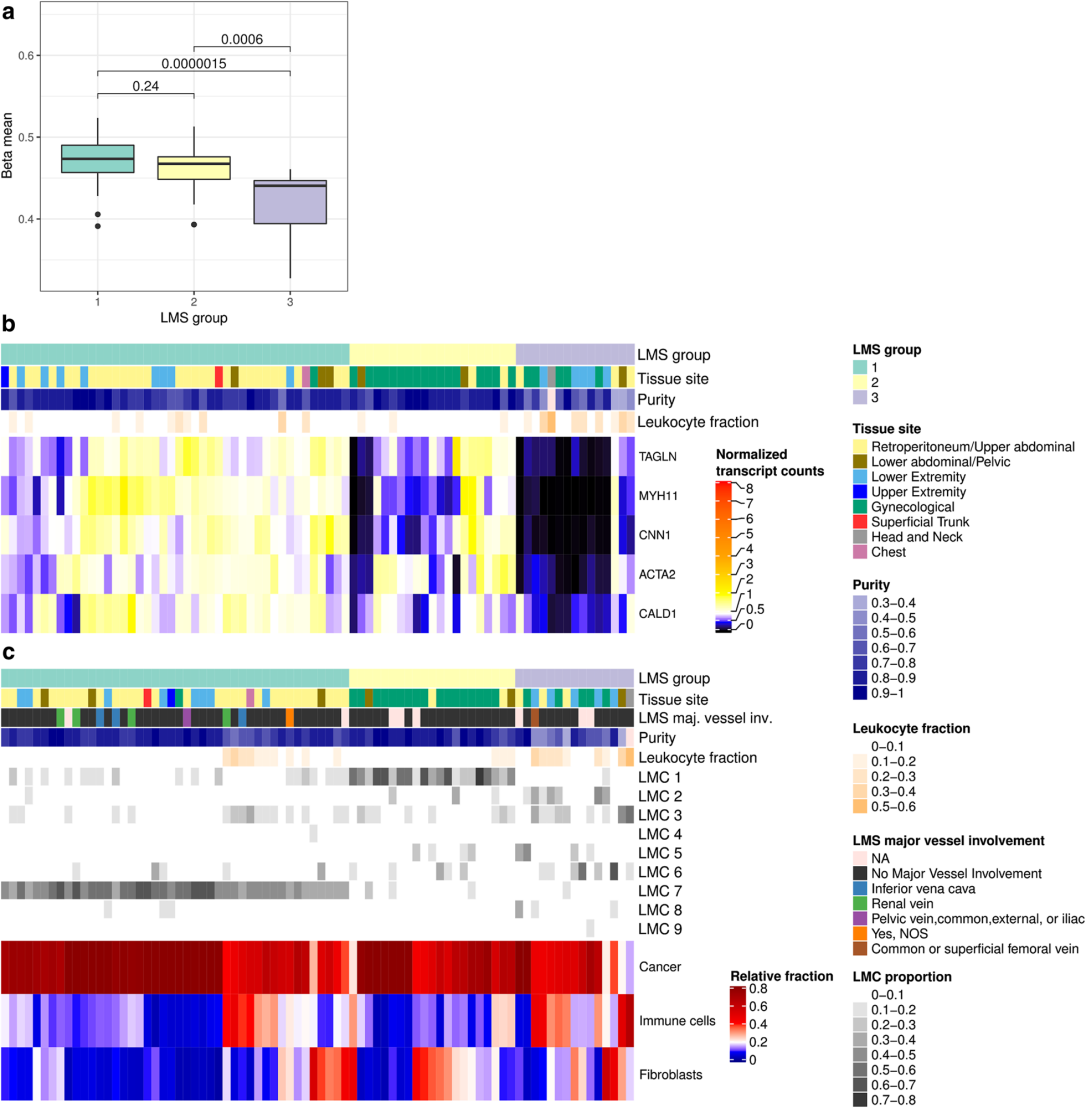
Comparison of DNA methylation and mRNA expression between LMS groups. **a** Global methylation differences between LMS subgroups. For each LMS sample, the mean methylation was calculated by averaging all available CpG probes. Groups were compared pairwise with the Wilcoxon test. **b** mRNA expression of known smooth muscle marker genes [11]. Samples were hierarchically clustered within the LMS groups in columns and genes were clustered in rows using the Euclidean distance metric and complete linkage. **c** Estimated relative cellular composition of the LMS groups based on MethylCIBERSORT [12]. The results suggest the presence of immune cells and fibroblasts in several samples across all LMS subgroups. Tumor purity correlated with estimated immune cell content, but not with fibroblast content (Pearson correlation purity—immune cell fraction − 0.82, Pearson correlation purity—fibroblast fraction -0.06). No association of major blood vessel involvement with any LMS group was observed

LMS group 3, comprising 15 cases had low proportions of both LMC7 and LMC1. We observed global DNA hypomethylation in these tumors compared to LMS groups 1 and 2 (Fig. 3a). The samples in this subgroup had high proportions for either LMC2, 3, 5 or 6. Gene set enrichment showed association with platelet-derived growth factor binding (*P* < 0.001) (LMC2), endocytosis (*P* < 0.006) and B cell receptor signaling pathway (*P* < 0.03) (LMC3), signaling pathways regulating pluripotency of stem cells (*P* < 0.017) (LMC5) and T cell receptor signaling pathway (*P* < 0.004) (LMC6) (Fisher's exact test and Benjamini−Hochberg correction).

In addition, we observed low expression of STLMS core signature genes, which might reflect dedifferentiation, lower muscle-specific activity or low tumor purity. This prompted us to compare the expression values of known smooth muscle marker genes [9–11] between the LMS groups. Figure 3b shows an overall low abundance of smooth muscle function-associated transcripts in LMS group 3.

Interestingly, the leukocyte fraction estimates based on methylation signatures by Abeshouse et al. (2017) showed enrichment in LMS group 3 cases compared to the other LMS groups (Fig. 3) [2]. By applying MethylCIBERSORT to methylation profiles, we estimated the relative abundance of immune cells in LMS cases. Notably, LMS group 3 showed a higher proportion of immune cells and fibroblasts, which likely also explains the observed lower tumor purity (Fig. 3c).

### Immune-cell infiltration differs substantially within and between sarcoma subtypes

We observed a strong correlation of the TCGA-SARC LMC3 proportion with the previously predicted leukocyte fraction (Pearson correlation 0.90) [2]. To further assess the association of LMC3 to immune cells, we deconvoluted methylation data from whole blood and sorted blood cell types [13]. Subsequent unsupervised hierarchical clustering of LMCs obtained from both datasets showed that TCGA-SARC LMC3 had a high similarity to different immune cell types (Additional file 1: Figure S4). In addition, the independent deconvolution of methylomes from the HIPO sarcoma cohort (HIPO LMC6) resulted in a methylation pattern with high similarity to TCGA-SARC LMC3 supporting the robustness of the deconvolution.

**Fig. 4:**
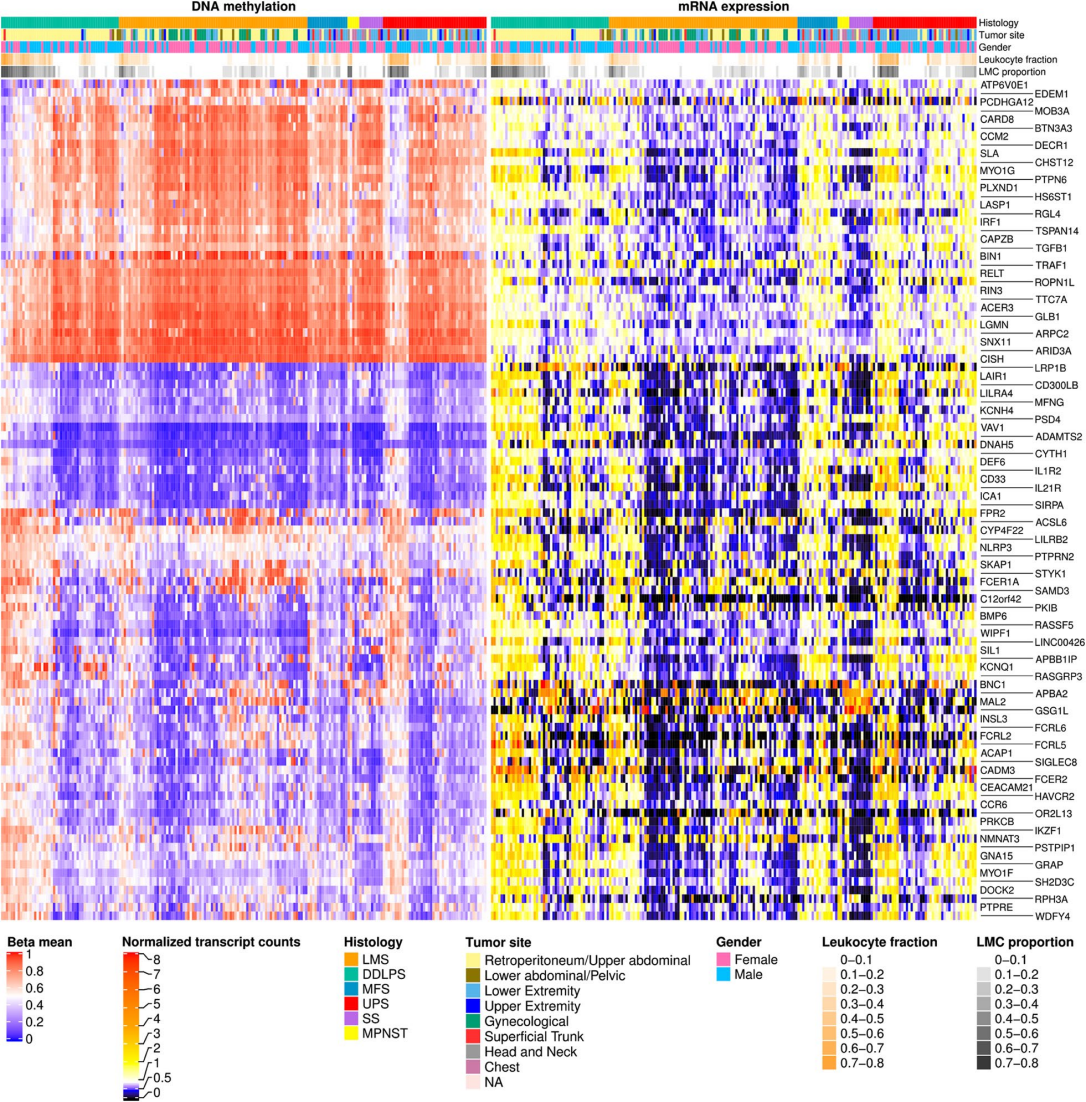
TIL core signature. The heatmap shows DNA methylation and mRNA expression for genes in samples with a high proportion of LMC3. Samples are clustered within the same histological subtype in columns, and genes are clustered in rows. The proportion of LMC3 strongly correlates with the predicted leukocyte fraction defined by [2]

Similar to the filtering approach described in the previous section, we enriched for genes with RNA expression correlated with methylation changes resulting in a set of 98 genes (Fig. 4). Of these genes, which we termed ‘TIL core signature’, 33 had hypomethylated and 65 had hypermethylated regions. A substantial fraction of the TCGA-SARC LMC3 signature genes were known immune cell markers. A comparison with a recently published list of cell-specific marker genes of tumor-infiltrating leukocytes (TILs) derived from pan-cancer data showed the presence of the B-cell marker *FCRL2* and the natural killer cell marker *IL21R* [14].

**Fig. 5:**
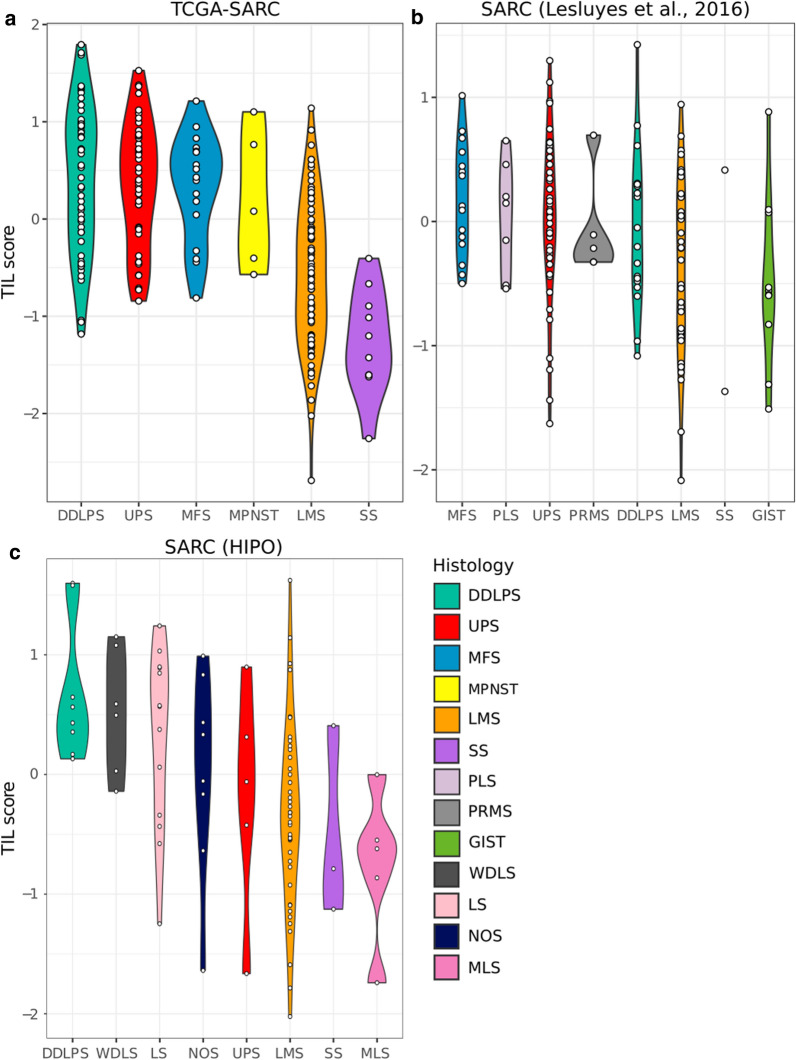
TIL score indicates varying degrees of immune cell infiltration in STS. TIL score of the sarcoma datasets. **a** TCGA-SARC, **b** Lesluyes et al. [15] and **c** in-house HIPO. The cohorts contain RNA sequencing data from 206, 135 and 89 samples, respectively. DDLPS, dedifferentiated liposarcoma; UPS, undifferentiated pleomorphic sarcoma; MFS, myxofibrosarcoma; MPNST, malignant peripheral nerve sheath tumor; LMS, leiomyosarcoma; SS, synovial sarcoma; PLS, pleomorphic liposarcoma; PRMS, pleomorphic rhabdomyosarcoma; GIST, gastrointestinal stromal tumor; WDLS, well-differentiated liposarcoma; LS, liposarcoma; NOS, sarcoma—not otherwise specified; MLS, myxoid liposarcoma

To get an estimate for the fraction of TILs in individual samples, we calculated a score based on the median expression of the TIL core signature genes for each sample (Fig. 5a). The results showed a high immune infiltration score for DDLPS and UPS, an intermediate score for LMS and a low score for SS. Additionally this demonstrated a high heterogeneity of TIL content within all subgroups except for SS, which generally had a low TIL score. A similar trend was observed in two independent sarcoma cohorts consisting of 224 sarcoma samples from twelve different subtypes (Fig. 5b, c) [15].

Further, to interrogate whether the immune cells in the tumor samples exhibited anti-tumor activity or merely originated from blood vessels in the tumor tissue, we calculated the correlation of TIL score with expression of two known cytolytic markers, granzyme A (*GZMA*) and perforin (*PRF1*) (Additional file 1: Figure S8) [16]. As RNA expression levels for both markers strongly correlated with the TIL score (Pearson correlation 0.72 and 0.78), we concluded that TCGA-SARC LMC3 captures a signal partly originating from cytotoxic T cells.

**Fig. 6:**
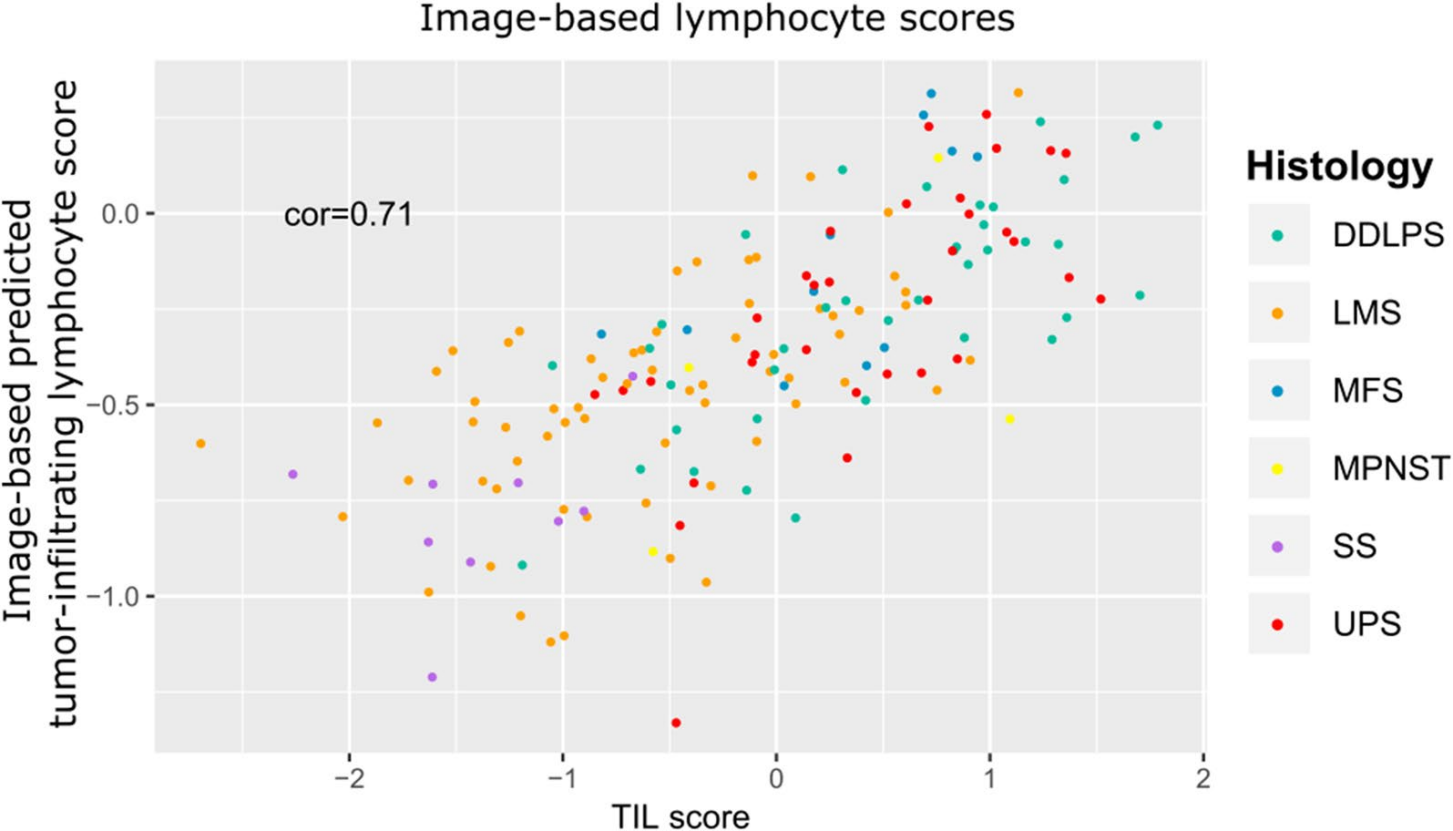
TIL score accords with image-based predicted tumor-infiltrating lymphocyte score. Scatterplot of image-based scores of tumor-infiltrating lymphocytes against TIL score for TCGA-SARC. The Pearson correlation coefficient is indicated in the top left corner. Colours indicate the histological subtypes

Next to test the robustness of the defined TIL scores we thought to integrate information from an independent assay and thus decided to utilize information from hematoxylin and eosin stained tumor slides of the same patients. For this purpose we used a recently published image-based study predicting the tumor-infiltrating lymphocyte content [17]. Our TIL scores for TCGA-SARC highly correlated with the predicted image-based tumor-infiltrating lymphocyte score (Pearson correlation 0.71), supporting the robustness of the TIL core signature [2, 17] (Fig. 6).

**Fig. 7:**
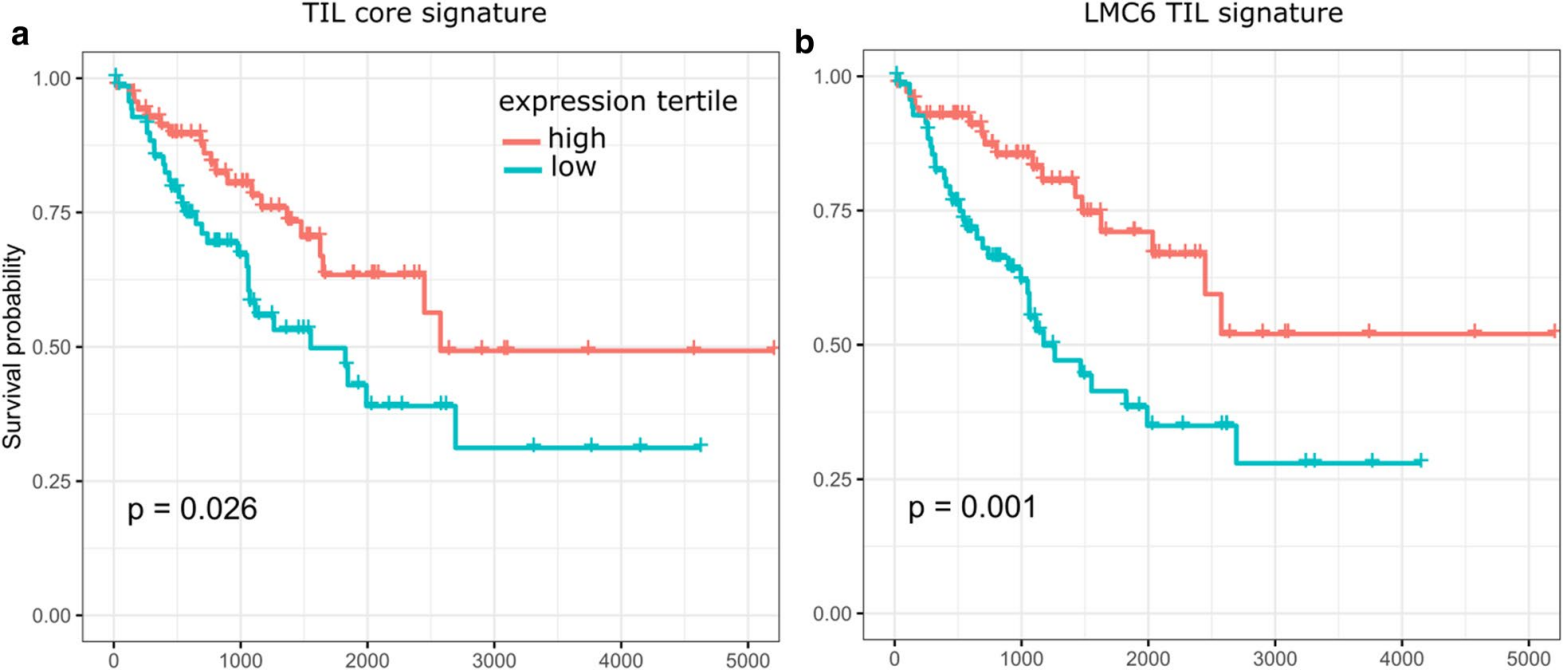
Kaplan–Meier analysis shows significant differences in overall survival between TIL groups. The overall survival of patients in the upper expression tertile was compared against patients in the lower tertile for the TCGA-SARC cohort using **a** the TIL core signature score or **b** the TIL score calculated from LMC6 signature genes as predictor

To investigate whether the TIL score is associated with clinical outcomes, we stratified the TCGA-SARC cohort into three equally sized groups of high, medium, and low TIL score. We then compared overall survival (OS) between the high and low TIL group (Fig. 7a). High expression of the immune gene signature resulted in improved OS within the whole sarcoma cohort (*P* = 0.026, log-rank test). Moreover, survival analysis based on the expression of single genes from the TIL core signature revealed *FCER1A* and *FCER2* as best predictors for OS (*P* = 0.021 and 0.006, log-rank test and Benjamini − Hochberg correction) (Additional file 1: Figure S9).

In addition to LMC3, a modest correlation was observed between LMC6 proportion and the predicted leukocyte fraction. Nevertheless, the mean mRNA expression for LMC6 signature genes showed a high correlation (Pearson correlation between mean LMC6 gene expression and leukocyte fraction 0.80, Additional file 1: Figure S10). Among other genes, immune cell markers in this component included *CD4*, *CD28* and *IL10*, indicating that this LMC captures methylation patterns from T cells. Notably, overall survival upon patient stratification based on LMC6 derived signature showed better separation compared to the TIL core signature (*P* = 0.001, log-rank test) (Fig. 7b). We also observed a higher fraction of promoter-resident CpG probes in LMC6 compared to all other LMCs (Additional file 1: Figure S6C).

### TILs are associated with specific genomic alterations in UPS and DDLPS

To find a potential mechanistic explanation for the varying degrees of TIL infiltration**,** we examined associations between the high and low TIL groups and their respective genomic alterations using Fisher’s exact test. We included somatic single nucleotide variations (SNVs), small insertions/deletions (indels), gene fusions and copy number aberrations (CNAs) in our analysis.

**Fig. 8:**
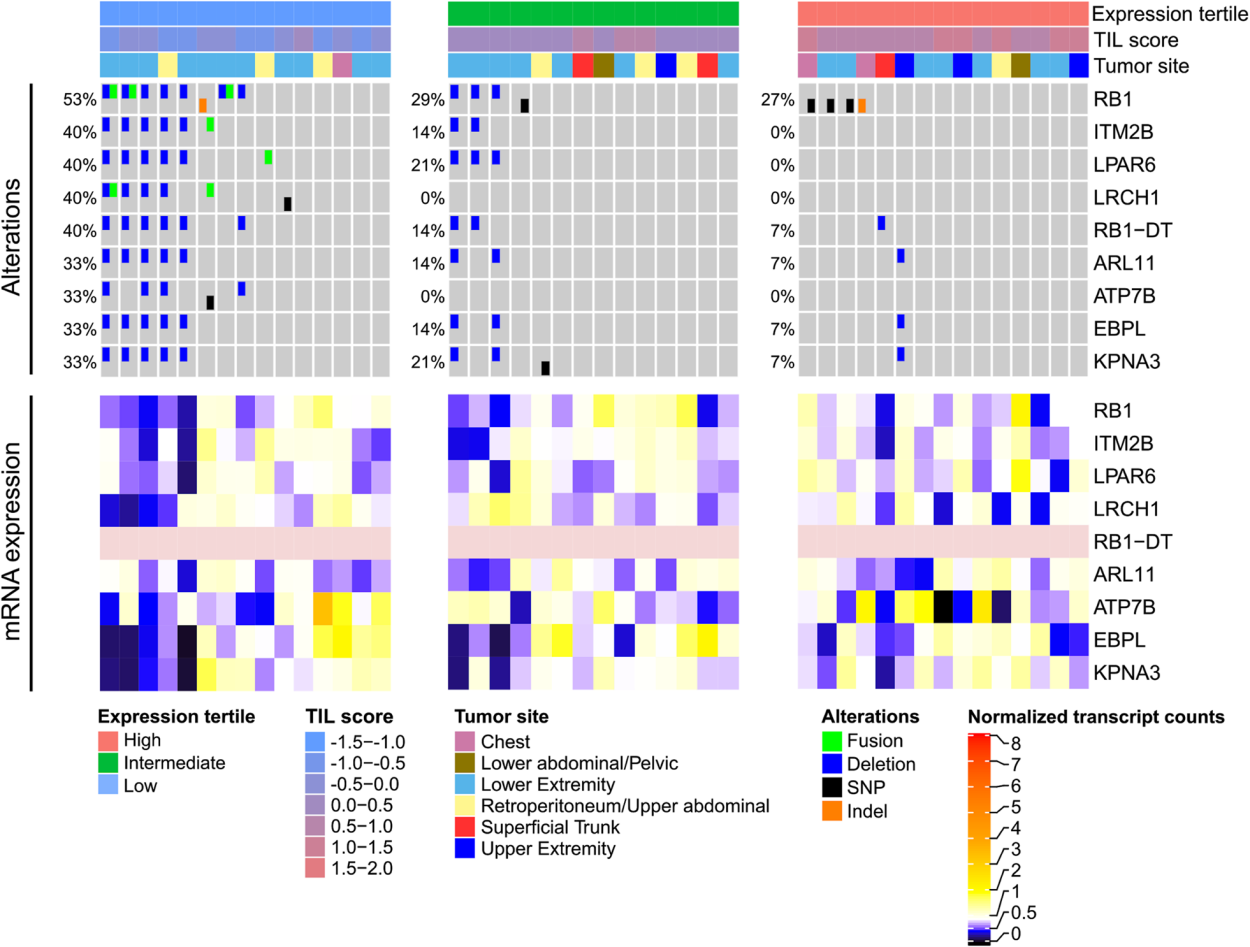
TILs are associated with a deletion of 13q14.2 in UPS. Samples were split into three equally sized groups based on their TIL score. The oncoprint shows deletions, fusions and SNVs enriched in the lower TIL score tertile for UPS. For each group and gene, the percentage of samples with at least one alteration is given. Additionally, the bottom heatmaps show matched gene expression data (pink: not available). With the exception of *ATP7B*, all of the deleted genes were located in the 13q14.2 region. The tumor suppressor gene *RB1* was deleted in seven out of 15 samples belonging to the low TIL group

In UPS, we found a deletion of 13q14.2 with a higher frequency in tumors with low TIL score compared to tumors with high TIL score (Fig. 8). The genes affected by the deletion events include *RB1*, *ITM2B, LPAR6*, LRCH1, *RB1-DT*, *ARL11*, *EBPL*, and *KPNA3*. *RB1* together with ARL11 were among the genes with the highest correlation between copy number and TIL score among all genes in the UPS cohort (copy number—TIL score Pearson correlation 0.46 and 0.43, respectively). In addition, we observed downregulation of genes affected as a result of 13q14.2 deletion. Overall, *RB1* expression was significantly lower in the low TIL group compared to the high TIL group in other sarcoma subtypes from the TCGA-SARC and Lesluyes et al. [15] cohorts (Additional file 1: Figure S11).

**Fig. 9:**
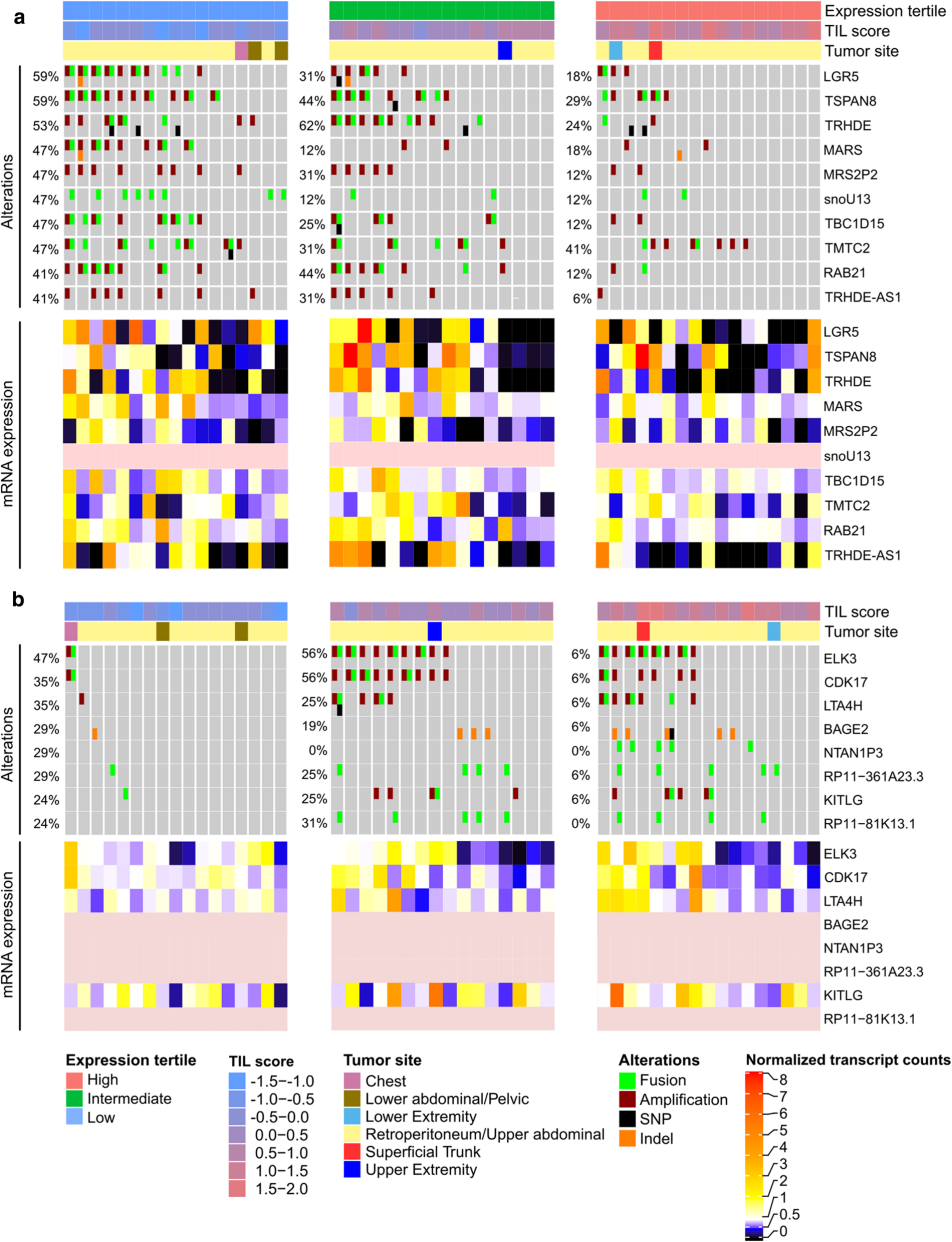
TILs are associated with specific copy number alterations and fusions in DDLPS. Samples were split into three equally sized groups based on their TIL score. The oncoprint shows genomic alterations enriched in the lower (**a**) and upper (**b**) TIL score tertile for DDLPS. In this sarcoma subtype, amplifications and fusions were most abundant. For each group and gene, the percentage of samples with at least one alteration is given. Additionally, the bottom heatmaps show matched gene expression data (pink: not available). In the low TIL group, 12q21.1 amplification affected the genes *LGR5, TSPAN8, TRHDE, RAB21, TBC1D15* and *TRHDE-AS1*, whereas an amplification of 12q23.1 including *ELK3, CDK17* and *LTA4H* was enriched in samples from high and intermediate TIL score expression tertiles

Next, we sought to investigate genomic alterations in DDLPS associated with the TIL groups. In the low TIL group, an amplification of chromosome 12q21.1 comprising the genes *LGR5*, *TSPAN8*, *TRHDE*, *RAB21*, *TBC1D15* and *TRHDE-AS1* was enriched (Fig. 9a). All of the genes had a high correlation between copy number and expression (copy number—normalized RNA expression Pearson correlations 0.56—0.87) hinting towards the functional impact of the amplification. The multipotent stem cell marker gene *LGR5* and *TRHDE* were significantly upregulated in the low TIL group compared to the intermediate and high TIL groups for DDLPS (*P* < 0.001 and < 0.03, Wilcoxon rank-sum test). We also detected fusion events with genes known in the context of cancer such as *LGR5-TSPAN8* in the low TIL group (Additional file 1: Table S2).

In the high TIL group, a strong enrichment for an amplification event of chromosome 12q23.1 was observed (Fig. 9b). The amplification status was highly correlated with the expression of the genes *ELK3*, *CDK17* and *LTA4H* located within this region (copy number—normalized RNA expression Pearson correlations 0.82, 0.67 and 0.87). Besides amplifications, gene fusions involving *ELK3,* a transcription factor with a multifaceted role in cancer and immune infiltration, were enriched in the medium and high TIL groups (Additional file 1: Table S2). The presence of *ELK3* fusion transcripts was confirmed in our in-house HIPO sarcoma dataset and was validated using Sanger sequencing for 5 cases (Additional file 1: Table S3).

We further found that *BAGE2*, a cancer testis antigen, harboured SNVs in five DDLPS samples in the high TIL group and one sample in the low TIL group, respectively.

## Discussion

Here, we present the first study that uses unsupervised methylation-based deconvolution to identify distinct methylation signatures within and across sarcoma subtypes. Prior studies on the identification of STS subtypes have mainly relied on differential gene expression [9, 10]. However, given the higher stability of DNA methylation over RNA expression [18], we chose to perform deconvolution on methylation data. Our novel approach enabled the discovery of unbiased profiles of methylation changes with altered gene expression, which were significantly associated with histopathological subtypes, tumor tissue localization and degree of immune cell infiltration. In particular, we identified components highly associated with STLMS, ULMS, SS and TILs. Contrarily, no distinct methylation patterns were found for DDLPS, UPS, MFS and MPNST, indicating a higher degree of heterogeneity among these tumor types.

Through unsupervised deconvolution of tumor methylomes, we identified three molecular subgroups for LMS based on molecular differences in DNA methylation and gene expression. We showed that LMS have different degrees of muscle specificity, which is high in STLMS-associated group 1, intermediate in ULMS-associated group 2, and low in LMS group 3. On the one hand, we could attribute differences in STLMS core signature expression to a lower purity in these tumors. The results from MethylCIBERSORT indicated the presence of immune cells in a fraction of samples belonging to all LMS subgroups, which was concordant with the estimated leukocyte fraction and tumor purity.

On the other hand, group 3 LMS tumors may represent some degree of dedifferentiation resulting in a less prominent smooth muscle phenotype. Furthermore, the dedifferentiated state in group 3 LMS might be linked to global hypomethylation, a mechanism frequently employed in cancer initiation and progression [19–21]. Our findings are in accordance with the prior studies using expression profiling to investigate subgroups in LMS [9, 10]. In addition, the prognostic immunohistochemical markers, MYLK and CASQ2, were also part of the derived STLMS core signature [21].

Chakravarthy et al. performed a pan-cancer methylation-based deconvolution of tumor samples and classified sarcomas as ‘immune cold’, characterized by low infiltrates of cytotoxic T-lymphocytes (CTLs) [12]. However, the notion that sarcomas are immune-quiescent tumors is challenged by an increasing number of studies [5, 22, 23].

In our study, we showed a varying degree of immune cell infiltration within and between sarcoma subtypes. Our results suggest that UPS and DDLPS on average have a higher immune cell infiltration compared to SS and LMS. Our findings are broadly in agreement with the TCGA-SARC study, where a high degree of macrophage infiltration in UPS/MFS and DDLPS and high score for CD8 positive cells was reported in DDLPS [2].

Results from the pioneer SARC028 clinical trial, where several metastasized STS subtypes together with bone sarcomas were treated with the anti-PD-1 antibody pembrolizumab, showed an 18% objective response rate, mainly coming from UPS and DDLPS patients [3]. A recent study by Keung et al., 2020, based on the pretreatment biopsies from patients enrolled in the aforementioned trial, reported higher densities of activated T cells as well as infiltration of tumor-associated macrophages in patients who responded to pembrolizumab [24]. The results show that only subgroups of patients with high immune cell infiltration might benefit from immunotherapy.

Furthermore, we found a strong correlation of TIL score with the expression of the cytolytic marker genes *GZMA* and *PRF1* indicating an abundance of CTLs and NK cells in these tumor samples. On the contrary, several genes with immunosuppressive effect such as inhibitory cytokines *IL10* and *TGFB1* and the cell surface receptor *HAVCR2* [25–27] were also expressed in the samples with high immune cell infiltration. Together, these proteins play an important role during T cell exhaustion, a condition frequently observed in the tumor microenvironment [28].

Among the genes belonging to the TIL core signature, expression of *FCER1A* and *FCER2* showed the highest association with overall survival in patients. These genes encode the Fc fragments of immunoglobulin epsilon (IgE) receptors FcεRI, highly expressed on mast cells and basophils [29], and FcεRII, found on the surface of various immune cells such as B and T cells, and also other cell types [30], respectively. In a recent study by Petitprez et al., B cells have shown to be a strong predictor of survival and response to PD1 blockade therapy in sarcoma [31]. Tumor-associated mast cells can have favourable or unfavourable effects on survival dependent on cancer type [32].

In summary, while there have been conflicting reports on the influence of immune cell infiltration on the clinical outcomes of sarcoma patients [33], our study indicates an overall beneficial effect of TILs on patient survival.

A limitation of our study is that an unbiased deconvolution is unable to completely disentangle the immune cell-associated component into contributions from different immune cell types based on their methylomes. LMC signature genes and corresponding GO and KEGG terms indicate that LMC3 captures a signal partly coming from B cells and LMC6 from T cells. To estimate the contributions from different immune cell types to the overall immune fraction, reference-based methods such as MethylCIBERSORT or CIBERSORT have been used [12, 34]. Here, a potential issue is that reference signatures were derived from cell lines, which may differ substantially from the cells in the tumor environment [35].

Previous studies have characterized the tumor microenvironment and immune profile of STS but there is limited knowledge about the specific immunogenicity in context of genomic alterations [5, 22, 23, 31].

In the low TIL UPS subgroup, we detected a 13q14.2 deletion including the tumor suppressor gene *RB1*. These results are in line with a recent pan-cancer analysis, where the authors reported a significant negative correlation between *RB1* deletion and their derived immune signature score [36]. Besides UPS, we also detected a consistent positive correlation between *RB1* expression and TIL score for LMS and DDLPS in two independent datasets (TCGA-SARC, Lesluyes et al. [15]). Previous studies have shown that retinoblastoma protein regulates the immune response by activating immune signalling pathways and its loss leads to decreased leukocyte recruitment resulting in tumor immune evasion [37–39]. *RB1* downregulation may present an immune evasion mechanism in sarcomas particularly of relevance in LMS, which are almost invariably characterized by loss of *RB1* function [40].

In the low TIL DDLPS subgroup, we observed a recurrent amplification of chromosome 12q21.1 harboring the multipotent stem cell marker *LGR5* and its corresponding upregulation. *LGR5* has been shown to positively regulate the Wnt signalling pathway, which is inversely correlated to B and T cell infiltration [41–44]. This relationship is concordant with our observation of a higher *LGR5* expression in the low TIL group.

In the high TIL DDLPS subgroup, there was an overrepresented amplification of chromosome 12q23.1. Amongst other genes, *ELK3*, a Ras-activated transcription factor from the ETS-family, is located within this region. In addition, we found novel *ELK3* fusion events in high and intermediate TIL DDLPS groups. In previous studies, *ELK3* upregulation resulted in increased metastatic behaviour of breast cancer and liver cancer stem cells by enhancing cell migration and invasion, whereas its suppression led to a reversal of the epithelial-mesenchymal transition in breast cancer cells [45–47]. We also observed frequent mutations of *BAGE2*, a cancer testis antigen, in the high TIL DDLPS group. Mutations in *BAGE2* have been hypothesized to play a role in immune evasion in osteosarcomas [48]. However, functional validations are required to test the role of these alterations in cancer with respect to TIL.

In summary, our unsupervised deconvolution of methylation data and subsequent integration with gene expression data revealed that STS exhibit varying degrees of immune cell infiltration, which is associated with clinical outcomes. Moreover, our results suggest that LMS can be stratified into three distinct subtypes based on methylation profiles. Finally, integration of genomics data unveiled key immune modulatory alterations associated with TIL infiltration. Overall, our study provides an important resource for patient stratification and predicting response and disease outcome to immune therapies.

## Methods

### Data availability and processing

We used the TCGA legacy data portal (https://portal.gdc.cancer.gov/legacy-archive) to download molecular data including level 2 Infinium Illumina HumanMethylation450 BeadChip (HM450K) array, level 3 whole-transcriptome RNA-sequencing data, level 2 non-silent somatic single nucleotide variations called by MuTect (v.1.1.6), and copy number variation data (segmented data from Affymetrix SNP array 6.0, level 3) of the SARC cohort. For gene expression analysis, we used the RSEM-quantified transcript counts, which were normalized within-sample to the fixed 75th percentile. To show relative differences in expression, the normalized counts for each gene were divided by its median expression in the cohort and subsequently log2-transformed. The 206 TCGA-SARC HM450k array samples were filtered for CpG probes with measurements available for all samples (394,363 probes). We further removed probes on the sex chromosomes and non-CpG probes.

DNA and RNA from the tumor specimen were isolated using the QIAamp DNA Mini Kit, the AllPrep DNA/RNA/Protein Mini Kit and the AllPrep DNA/RNA/miRNA Universal Kit (Qiagen). The Generead DNA FFPE kit and the QIAamp DNA FFPE Tissue Kit (both Qiagen) were used for extracting the nucleic acids from formalin-fixed paraffin embedded (FFPE) samples. Quality control and quantification steps were done using a Qubit Fluorometer (Life Technologies) and the E-Gel Agarose Electrophoresis System (Invitrogen) or the 2200 TapeStation system (Agilent). The methylation analysis was carried out according to the manufacturer's specifications (Illumina Infinium HD methylation assay reference guide and for FFPE samples Illumina Infinium-HD-FFPE-assay-reference-guide).

Preprocessing of in-house sarcoma methylation data (MASTER) [53] was done with RnBeads (v.2.0.0) in R (v.3.5.1) [49]. First, raw idat files generated from Illumina MethylationEPIC BeadChip microarrays were imported into R using the function 'rnb.execute.import'. We then removed all probes overlapping SNPs ('rnb.execute.snp.removal'), probes with detection p-values above 0.05 ('rnb.execute.greedycut'), probes located on sex-chromosomes ('rnb.execute.sex.removal'), probes outside of CpG context ('rnb.execute.context.removal'), and probes with standard deviation below 0.005 ('rnb.execute.variability.removal'). Normalization was performed using ‘BMIQ’ together with ‘enmix.oob’ as a background correction method using the function 'rnb.execute.normalization'. We kept all samples from sarcoma subtypes occuring at least twice in the dataset resulting in 56 samples from 13 different histopathological entities.

Four RNA-seq datasets were additionally included for validation of the results from TCGA-SARC: we downloaded RNA-seq data from a second sarcoma cohort with 135 samples [15] (GEO: GSE71119) and 60 HM450k array samples with blood cells from magnetic-activated cell sorting [13] (GEO: GSE35069). Eventually, two in-house cohorts were used for validation of the TIL score (HIPO28) and fusion validations (HIPO21), respectively.

RNA sequencing libraries were prepared using the TruSeq RNA Sample Preparation Kit v2 (Illumina), normalized to 10 nM, pooled to 11-plexes, and clustered on a cBot system (Illumina) to a final concentration of 10 pM with a spike-in of 1% PhiX Control v3 (Illumina). Paired-end sequencing (2 × 101 bp) was carried out with a HiSeq 2000 instrument (Illumina). Reads were mapped with STAR (version 2.3.0e). 1000 Genomes reference sequence with GENCODE version 17 transcript annotations was used for building the index. For alignment, the following parameters were used: alignIntronMax 500,000, alignMatesGapMax 500,000, outSAMunmapped Within, outFilterMultimapNmax 1, outFilterMismatchNmax 3, outFilterMismatchNoverLmax 0.3, sjdbOverhang 50, chimSegmentMin 15, chimScoreMin 1, chimScoreJunctionNonGTAG 0, chimJunctionOverhangMin 15. The output was converted to sorted BAM files with SAMtools, and duplicates were marked with Picard tools (version 1.90).

### Detection of gene fusions

Using our in-house pipeline Arriba (v0.8) [54], high-confidence gene fusion predictions were extracted from chimeric alignments produced by STAR. Arriba removes recurrent alignment artifacts, transcript variants which are also observed in normal tissue, or a low number of supporting reads relative to the overall number of predicted events in a gene, and reads with low sequence complexity as well as events with short anchors or breakpoints in close proximity. The fusions were filtered for genes, which occur in at most 20 samples and have supporting split reads (split_reads1 + split_reads2 > max(1, discordant_mates/10)).

### Fusion validation assays

Selected fusions were validated by the Center for Molecular Pathology at the Institute of Pathology of the Heidelberg University Hospital using orthogonal techniques such as Sanger sequencing.

### Non-negative matrix factorization with MeDeCom

We used reference-free non-negative matrix factorization of methylation data implemented in the MeDeCom package to recover biologically meaningful methylation patterns [7].

Prior to deconvolution with MeDeCom (v.0.2), beta values of HM450k array CpG probes were averaged over a window size of 5 kb, referred to as windows to decrease the size of the input matrix and to cover all available genomic regions. Only windows with at least one CpG probe were kept. For TCGA-SARC, MeDeCom was run with K = 9.. 11, λ = 10^−5^.. 1, maximum 300 iterations, 10 random initializations, and tenfold cross-validation. For downstream analysis, K = 9 latent methylation components (LMCs) and λ = 0.01 was chosen. For the blood cell dataset, we ran MeDeCom with K = 5.. 20 and used 13 LMCs and λ = 0.001 for further analysis.

### Extracting LMC-specific hypo-/hypermethylated regions

To extract windows which are LMC-specific we categorized each LMC into hypo- and hypermethylated windows. For each window we compared the deconvoluted beta values in each LMC: a window was categorized hypomethylated for a LMC if the deconvoluted beta value of the window was smaller by at least 0.2 compared to the beta values of all other LMCs. Analogously we applied the definition to extract hypermethylated windows.

### Correlation of hypo-/hypermethylated windows to gene expression

We intended to enrich the hypo- and hypermethylated LMC-specific windows for functional regions. Therefore, we correlated the beta values of LMC specific hypo- and hypermethylated windows using the expression values of the gene associated with the window. In detail, beta values from the CpG probes lying within the LMC-specific genomic windows were extracted and averaged by their associated gene using annotations from the IlluminaHumanMethylation450kanno.ilmn12.hg19 package (v.0.6.0) in R. We further filtered the genes with matched methylation and mRNA expression values by applying a correlation threshold of ± 0.3 using Pearson correlation. This workflow thus led to four categories of windows:

- Hypomethylated/negatively correlated
- Hypermethylated/negatively correlated
- Hypomethylated/positively correlated
- Hypermethylated/positively correlated

To derive signature gene sets for each LMC, we filtered for correlation patterns a) and d).

### Proportion and methylation-mRNA expression heatmaps

For a clear representation of subtype associations, samples were hierarchically clustered within each histological subtype using the Euclidean distance metric and complete linkage. To show methylation and corresponding mRNA expression values, we averaged beta values from LMC-specific CpG probes associated with each gene and calculated their relative mRNA expression as described in “Data availability and processing”. Genes were clustered based on their methylation values using the Euclidean distance metric and complete linkage.

### TIL score

The TIL score was calculated for each sample i as the median of log-normalized mRNA expression values from all LMC3-specific genes:

$$ TIL_{i} = median\left( {A_{i} } \right),\;  with\; A_{i} = {\text{U}}_{j }\, log_{2} \left( { \frac{{count_{i,j} }}{{median(count_{j} ) + 0.01}}} \right) \quad for\;  j = \left( {1...n} \right) $$

where i is the sample and j is the gene from the LMC3-specific signature containing n genes in total. For gene counts, normalized RSEM-quantified transcript counts (TCGA-SARC) or FPKM values (GSE71119) were used. Thus, assuming count ≫ 1, a value of 0 is equivalent to the TIL score of a sample being identical to the median TIL score for the cohort.

### Survival analysis

We investigated the association of signature gene expression with overall survival. For Kaplan–Meier survival analysis, the samples were grouped into three equally sized groups by TIL score or the expression level of single LMC3 and 6 signature genes. The 33% of samples with the highest score/expression were compared to the 33% of samples with the lowest score/expression. *P* values were calculated using the log-rank test. The log-rank *P* values for single signature genes were adjusted for multiple testing using the Benjamini–Hochberg method. We additionally performed univariable Cox regression with TIL score or the expression values of a single gene from LMC 3 or 6 as predictor.

### Identification of TIL group specific genomic alterations

To gain insight into the reasons for variable TIL abundance in the tumor samples, we performed an exploratory analysis of the differences in their genomic alterations. To this end, we grouped the samples for each subtype according to the TIL score into tertiles. In the comparison of genomic alterations, we included non-silent somatic single nucleotide variations, small insertions/deletions, deletions and amplifications, as well as fusion events. For each alteration type and gene, the number of occurrences in the upper TIL score tertile was compared to the number of occurrences in the lower TIL score tertile using Fisher’s exact test. All genes with a *P* < 0.2 in at least one of the compared alteration types were retained. Eventually, *P* values were adjusted for genes with events in multiple alteration types using Fisher’s method implemented in the ‘sumlog’ function from the metap package in R. Likewise, genes with *P* < 0.2 after adjustment were retained. For clarity, we included a maximum of ten genes with the lowest *P* values in the oncoprints. Since all genes in the dataset were tested, the results may include false positives arising from multiple testing and should therefore be regarded as exploratory.

In the oncoprints, genes were sorted based on the number of samples with at least one alteration within the TIL group of interest. Samples were sorted within each TIL group to show mutual exclusivity as implemented in the oncoPrint function of the ComplexHeatmap package (v.1.99.5) in R.

### LMS subgroups

We assigned the LMS cases to three groups based on their LMC proportions. Samples with proportion < 0.2 in both LMC1 (ULMS-associated) and LMC7 (STLMS-associated) were defined as LMS group 3. LMS group 1 was defined as LMC7 > LMC1 and LMC7 > 0.2, and LMS group 2 as LMC1 > LMC7 and LMC1 > 0.2.

### Gene ontology analysis

For gene ontology enrichment analysis, the online tool Enrichr 3 was used (queried on 2019/01/18) [50]. For determination of significance, the tool applies Fisher’s exact test, and subsequent *P* value adjustment for multiple testing with the Benjamini–Hochberg method. Gene-set libraries were employed from the Gene Ontology 2018 and KEGG 2016 databases [51, 52].

### Cibersort

As we noticed different tumor purities in the LMS subgroups, we applied MethylCIBERSORT deconvolution on the methylation data to get an estimate of the frequency of cell types in these tumor samples [12, 34]. We used the sarcoma signature matrix provided in the MethylCIBERSORT package in R, which contains methylation signatures for immune cells, fibroblasts and cancer cells from cell lines. The CIBERSORT algorithm was run without prior quantile normalization and 1,000 permutations. Subsequently, we summed up the resulting relative fractions from all immune cell types to allow a comparison of the overall proportion of immune cells, fibroblasts and sarcoma cells in each sample.

### Predictions of tumor-infiltrating lymphocytes from histopathology image slides

Predictions of tumor-infiltrating lymphocyte abundances from tissue slides were obtained from [17]. For each sarcoma sample the mean prediction score across all image tiles was calculated and used for comparison with the RNA-based TIL score.

### Assignment of probes to promoter, intragenic and intergenic regions

Gene annotations were extracted from the TxDb.Hsapiens.UCSC.hg19.knownGene annotation package (v.3.2.2) in R. We defined promoters as the region from 1000 bp upstream to 100 bp downstream of the transcription start site.

## Supplementary Information

**Additional file 1.** Additional figures.

**Additional file 2.** Additional table.

## Data Availability

RNA sequencing and data from the validation cohort project were deposited at EGA (https://www.ebi.ac.uk/ega/datasets/EGAD00001003828) under accession number EGAD00001003828 (LMS-RNAseq samples). Methylation array data was deposited at EGA under accession number EGAS00001005262. Code used in the analysis is available as R Notebook at https://bitbucket.org/simon_mt/sarcoma_deconvolution/
