# Correction to: Deconvolution of sarcoma methylomes reveals varying degrees of immune cell infiltrates with association to genomic aberrations

**DOI:** 10.1186/s12967-022-03316-8

**Published:** 2022-04-02

**Authors:** Malte Simon, Sadaf S. Mughal, Peter Horak, Sebastian Uhrig, Jonas Buchloh, Bogac Aybey, Albrecht Stenzinger, Hanno Glimm, Stefan Fröhling, Benedikt Brors, Charles D. Imbusch

**Affiliations:** 1 Division of Applied Bioinformatics, German Cancer Research Center (DKFZ), Heidelberg, Germany; 2 Division of Translational Medical Oncology, National Center for Tumor Diseases, German Cancer Research Center, Heidelberg, Germany; 3 Faculty of Biosciences, Heidelberg University, Heidelberg, Germany; 4 Institute of Pathology, University Hospital Heidelberg, Heidelberg, Germany; 5 German Cancer Consortium (DKTK), German Cancer Research Center (DKFZ), Heidelberg, Germany; 6 Department of Translational Medical Oncology, NCT Dresden, Dresden, Germany; 7 University Hospital Carl Gustav Carus, Technical University Dresden, Dresden, Germany

## Correction to: J Transl Med (2021) 19:204 https://doi.org/10.1186/s12967-021-02858-7

Following publication of the original article [1], the authors identified an error in the affiliation 2 for the authors. The incorrect and correct affiliation information is shown below. The author group has been updated above and the original article [1] has been corrected. Also, affiliation 2 should have been removed as one of the affiliations of the author Benedikt Brors:

**Incorrect**

- 2 Translational Oncology, National Center for Tumor Diseases, German Cancer Research Center, Heidelberg, Germany
- Benedikt Brors affiliations—1,2,5

**Correct**

- 2 Division of Translational Medical Oncology, National Center for Tumor Diseases, German Cancer Research Center, Heidelberg, Germany
- Benedikt Brors affiliations—1,5
