# Supplementary material for: Deconvolution of sarcoma methylomes reveals varying degrees of immune cell infiltrates with association to genomic aberrations
Source: J Transl Med. 2021 May 12;19:204. doi: 10.1186/s12967-021-02858-7 (PMC8117561; doi:10.1186/s12967-021-02858-7)
Supplement: Supplementary file 1 — Additional file 1. Additional figures. [file 12967_2021_2858_MOESM1_ESM.docx]

# Additional Figures

####
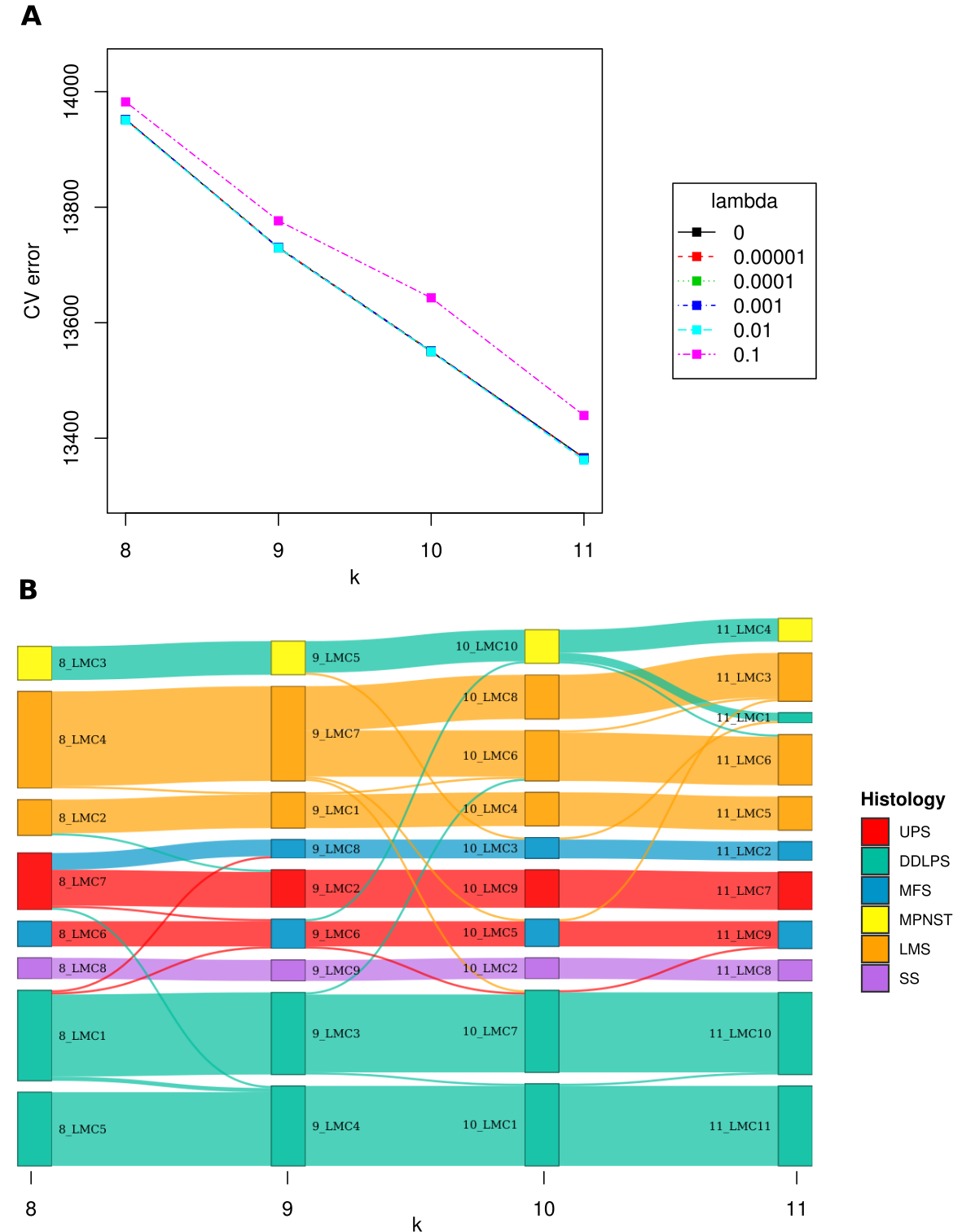


**Additional Figure S1. Cross-validation error and river plots for selection of appropriate deconvolution parameters.** The methylation data was decomposed into nine LMCs, based on the stability of the obtained methylation profiles with increasing numbers of LMCs and interpretability of the deconvolution results. **A** Cross-validation error from MeDeCom deconvolution with different numbers of LMCs (k) and λ. λ = 0.01 was chosen for further analysis. **B** River plot showing main sample subtype associations for deconvolutions with increasing numbers of LMCs. Each sample was assigned to the LMC with the highest proportion. The size of the nodes and links corresponds to the number of samples. The colour of the nodes represents the histological subtype with the highest mean proportion for the component. Links are coloured according to the subtype, which contributes most samples to the reassignment.


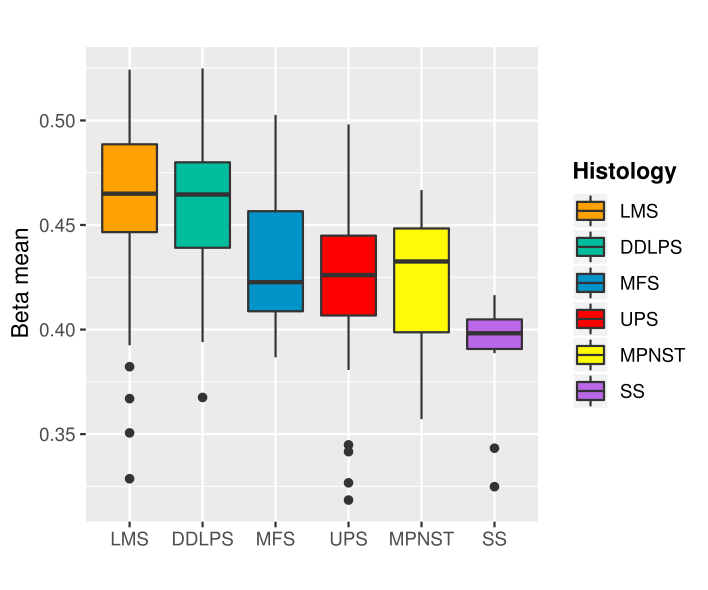


**Additional Figure S2. Global average methylation of sarcoma subtypes.** For each sample, the mean methylation was calculated by averaging all available CpG probes. The epigenome-wide comparison shows hypomethylation of SS compared to other sarcoma subtypes.


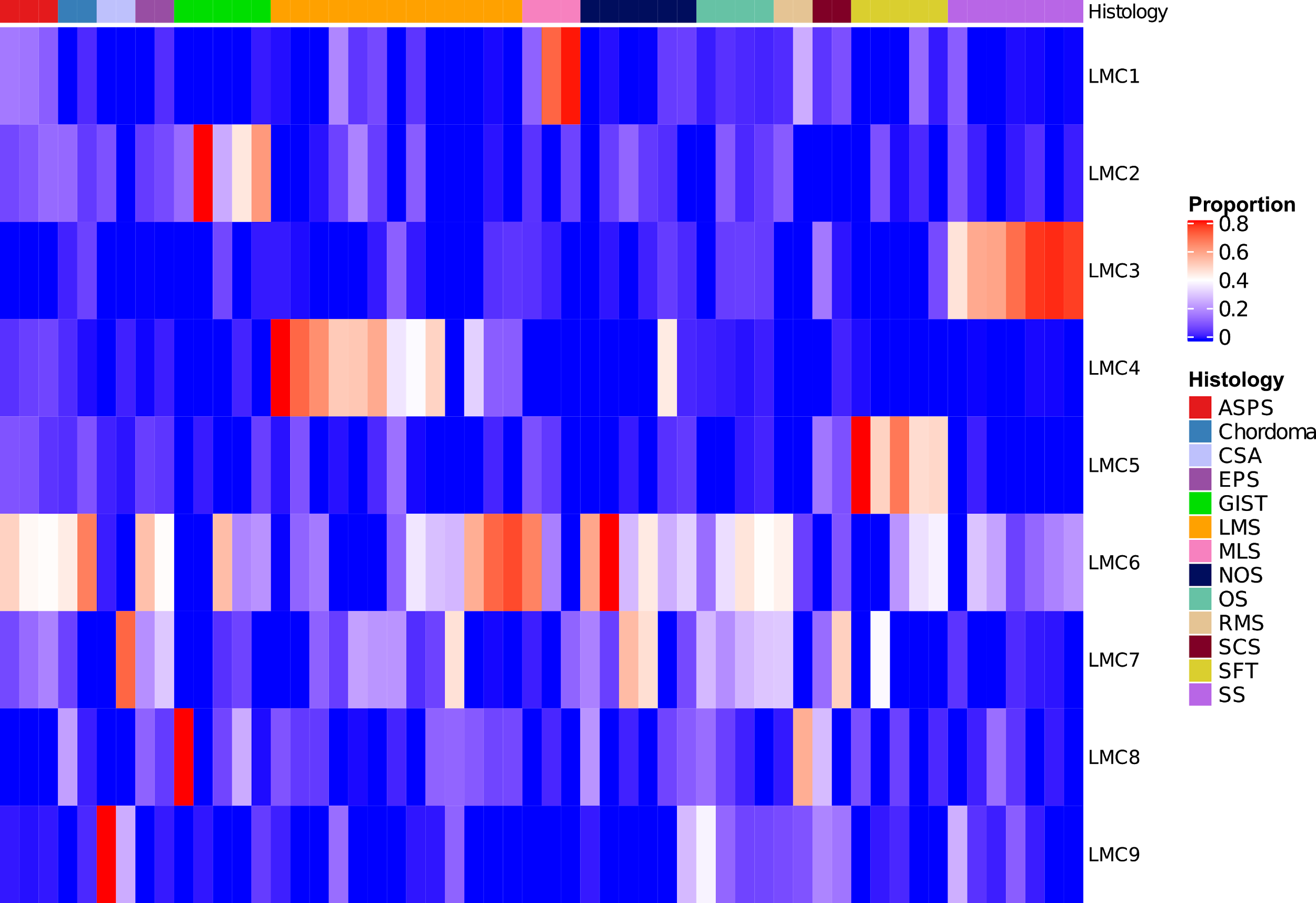


**Additional Figure S3. Deconvolution of independent sarcoma methylation dataset confirms identification of subtype-associated methylation patterns**. Proportions for the deconvolution of in-house SARC (HIPO) methylation data using 9 LMCs and λ = 0.01 are shown. Unsupervised deconvolution with MeDeCom resulted in several methylation patterns associated with distinct sarcoma subtypes. ASPS, alveolar soft part sarcoma; CSA, chondrosarcoma; EPS, epithelioid sarcoma; GIST, gastrointestinal stromal tumor; LMS, leiomyosarcoma; MLS, myxoid liposarcoma; NOS, sarcoma - not otherwise specified; OS, osteosarcoma; RMS, rhabdomyosarcoma; SCS, spindle cell sarcoma; SFT, solitary fibrous tumor; SS, synovial sarcoma.


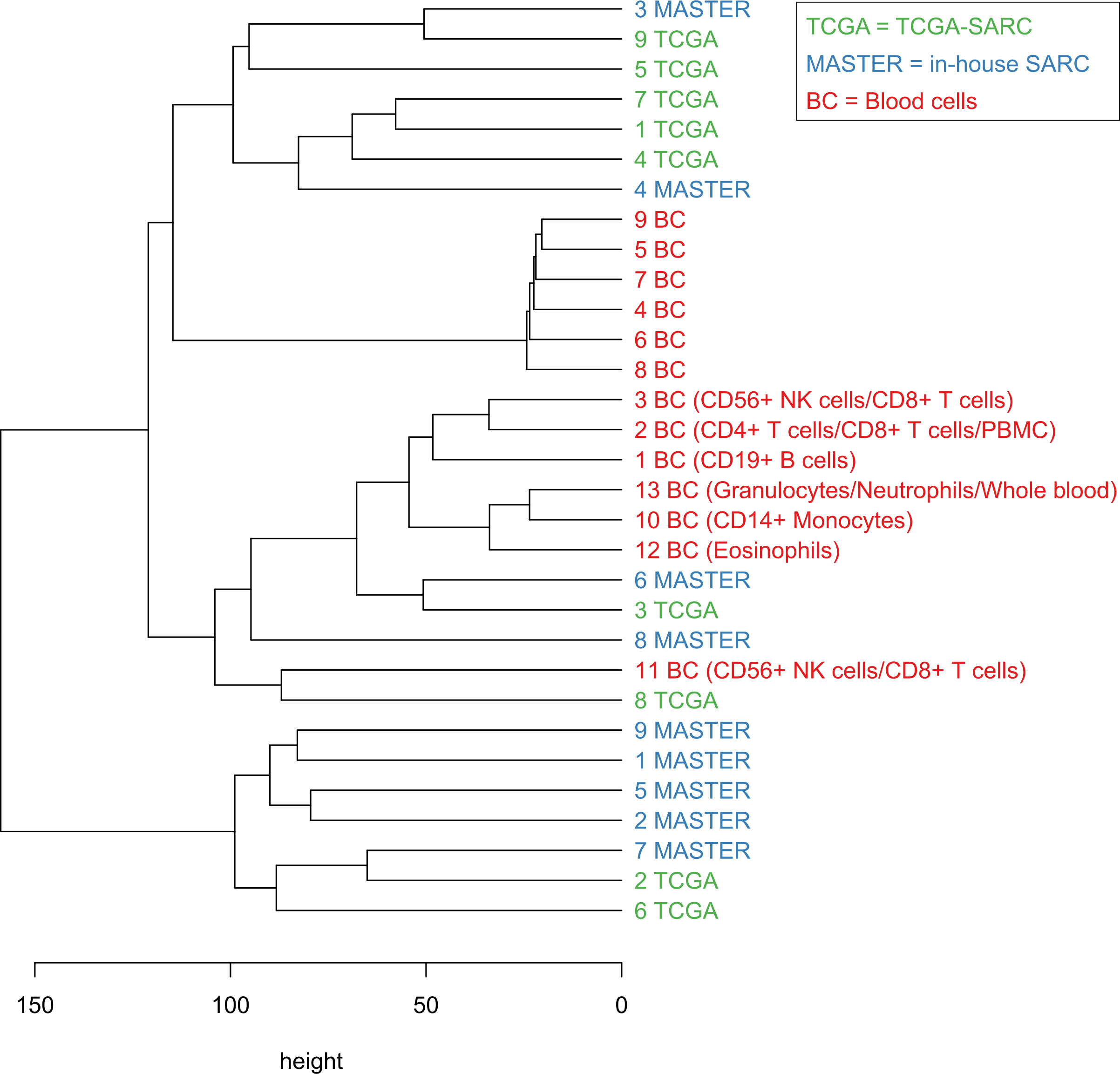


**Additional Figure S4. Hierarchical clustering of LMCs obtained from the deconvolution of TCGA-SARC, in-house SARC and blood cells.** Unsupervised hierarchical clustering of LMCs obtained by MeDeCom deconvolution of sarcoma (TCGA, 9 LMCs, MASTER, 9 LMCs) and blood cells (BC, 13 LMCs). Clustering was performed on the deconvoluted beta values of common windows in all datasets using Euclidean distance and complete linkage. Associated immune cell types for each BC LMC are indicated in brackets. TCGA LMC3 and MASTER LMC6 have a high similarity with the BC LMCs. LMCs associated with sarcoma subtypes cluster together, i.e., SS-associated TCGA LMC9 and MASTER LMC3 and LMS-associated TCGA LMCs 1 and 7 and MASTER LMC4. BC LMCs 4 – 9 are patient-specific.


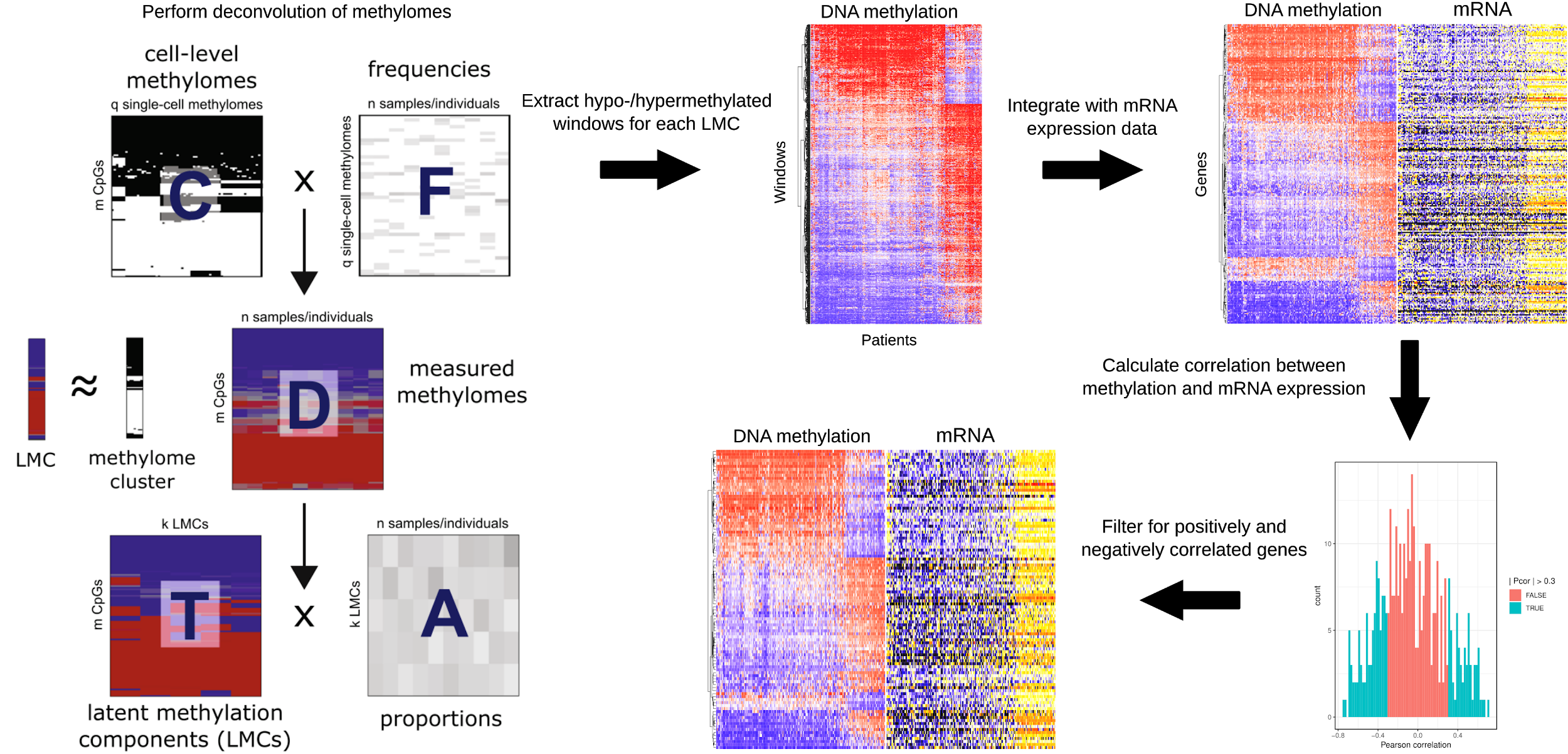


**Additional Figure S5.** Workflow used for extraction of genes with LMC-specific methylation and correlation to gene expression. Following deconvolution with MeDeCom, variably methylated windows were extracted for each LMC [7]. Integration of mRNA expression data allowed a subsequent filtering for genes based on correlation between DNA methylation and mRNA expression.


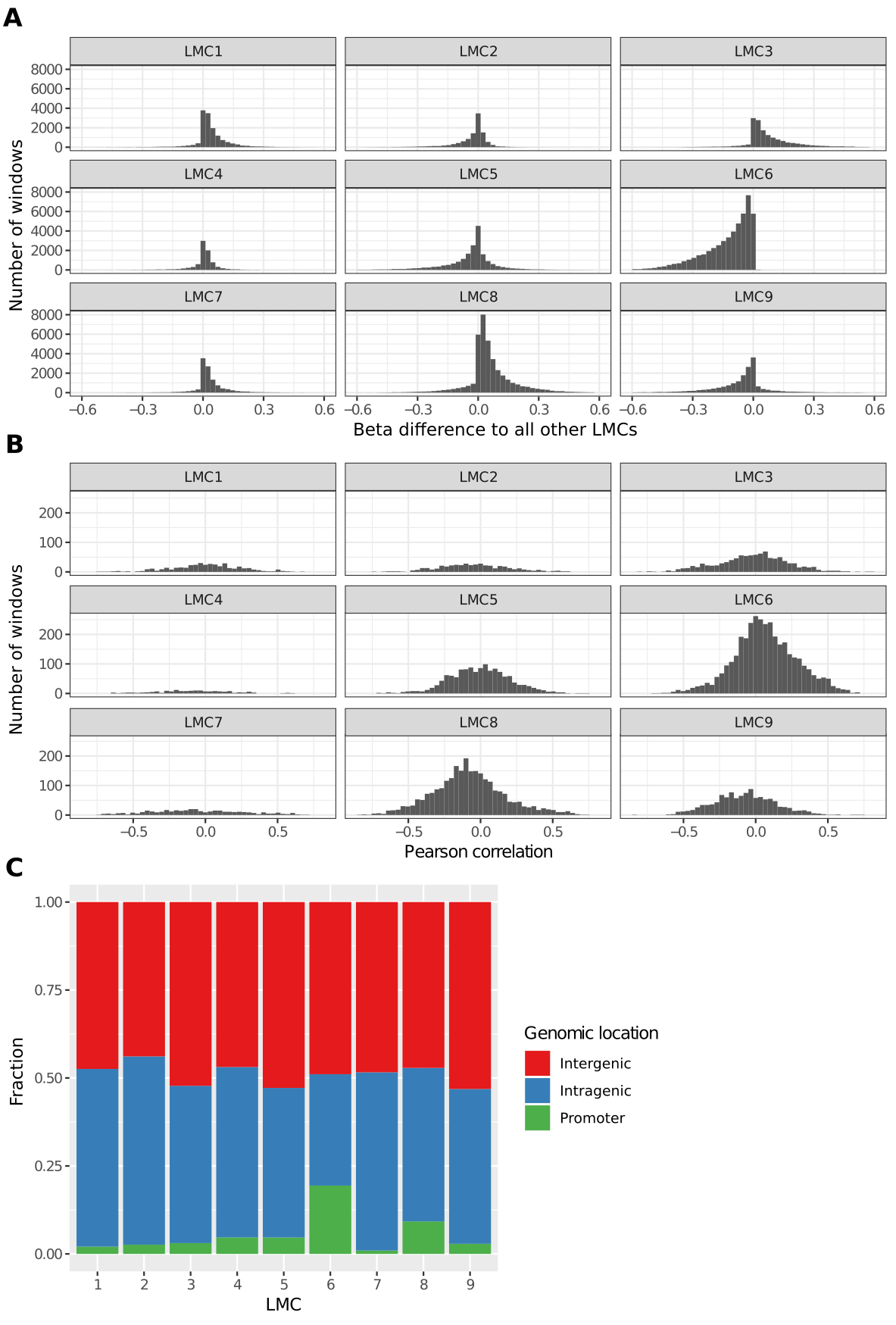


**Additional Figure S6. Characteristics of LMC-specific regions. A** Distribution of LMC-specific hypo- and hypermethylated genomic regions. For each LMC, tiles were binned by their minimum absolute difference of their LMC score compared to all other LMCs in the decomposition. The histogram shows the number of tiles in each bin, which were either hypo- or hypermethylated compared to all other LMCs. The x-axis was cropped at [-0.6, 0.6]. **B** Correlation between DNA methylation and mRNA expression for LMC-specific genes. CpG probes within LMC-specific hypo- and hypermethylated tiles were associated with genes, and subsequently correlated to the mRNA expression using the Pearson correlation coefficient. The histogram depicts the distribution of correlation coefficients. **C** Relative proportion of CpGs from LMC-specific tiles in promoter, intragenic or intergenic regions based on their genomic location.


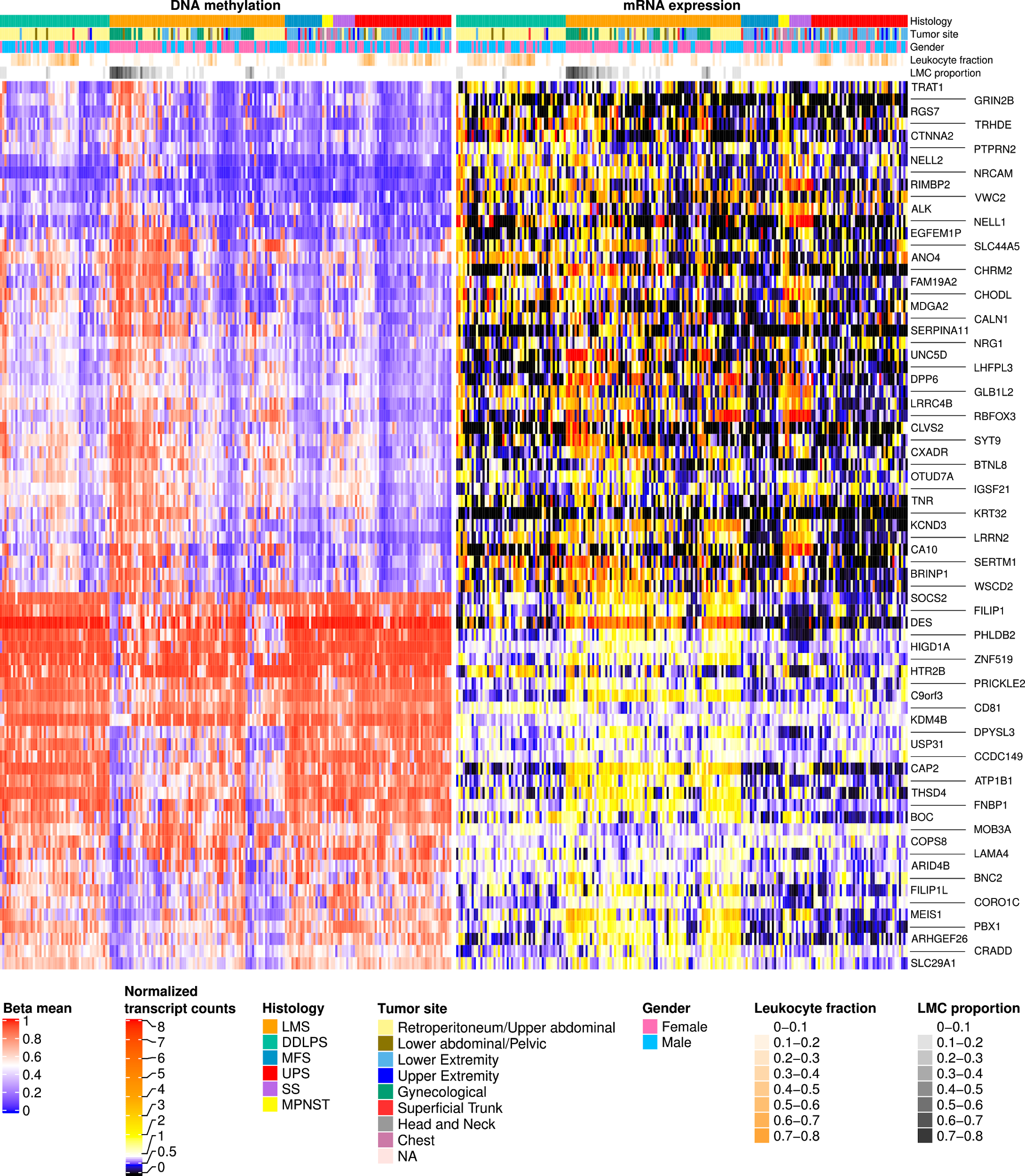


**Additional Figure S7. LMC1 gene signature associated with ULMS.** The heatmaps show DNA methylation and corresponding mRNA expression for upregulated genes, in samples with a high proportion of LMC1. Samples are clustered within the same histological subtype in columns, and genes are clustered in rows. Compared to the STLMS-associated LMC7 signature genes, the LMC1-specific genes have a lower association with ULMS.

####


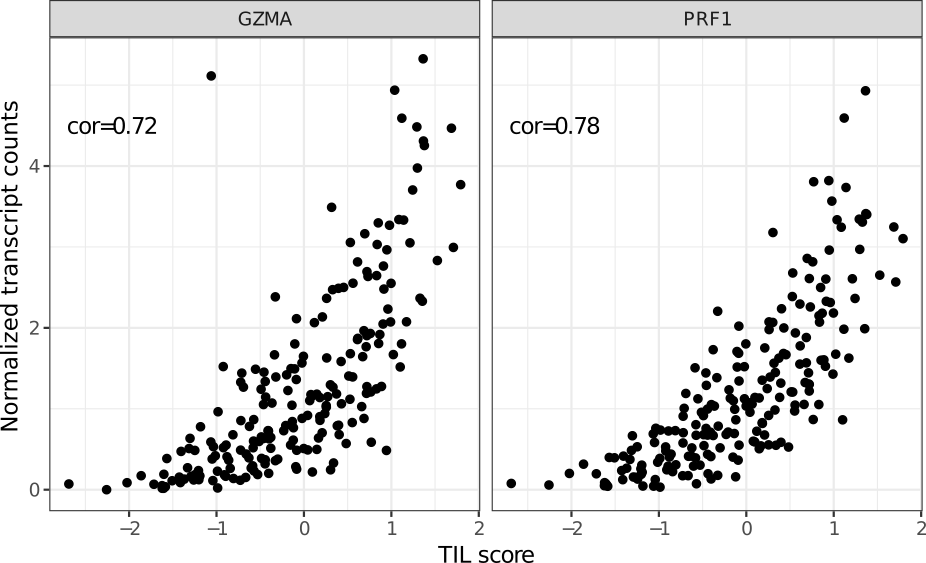


**Additional Figure S8. TIL score correlates with cytolytic activity.** Scatterplots of normalized GZMA or PRF1 expression against TIL score. The Pearson correlation coefficients are indicated in the top left corner.


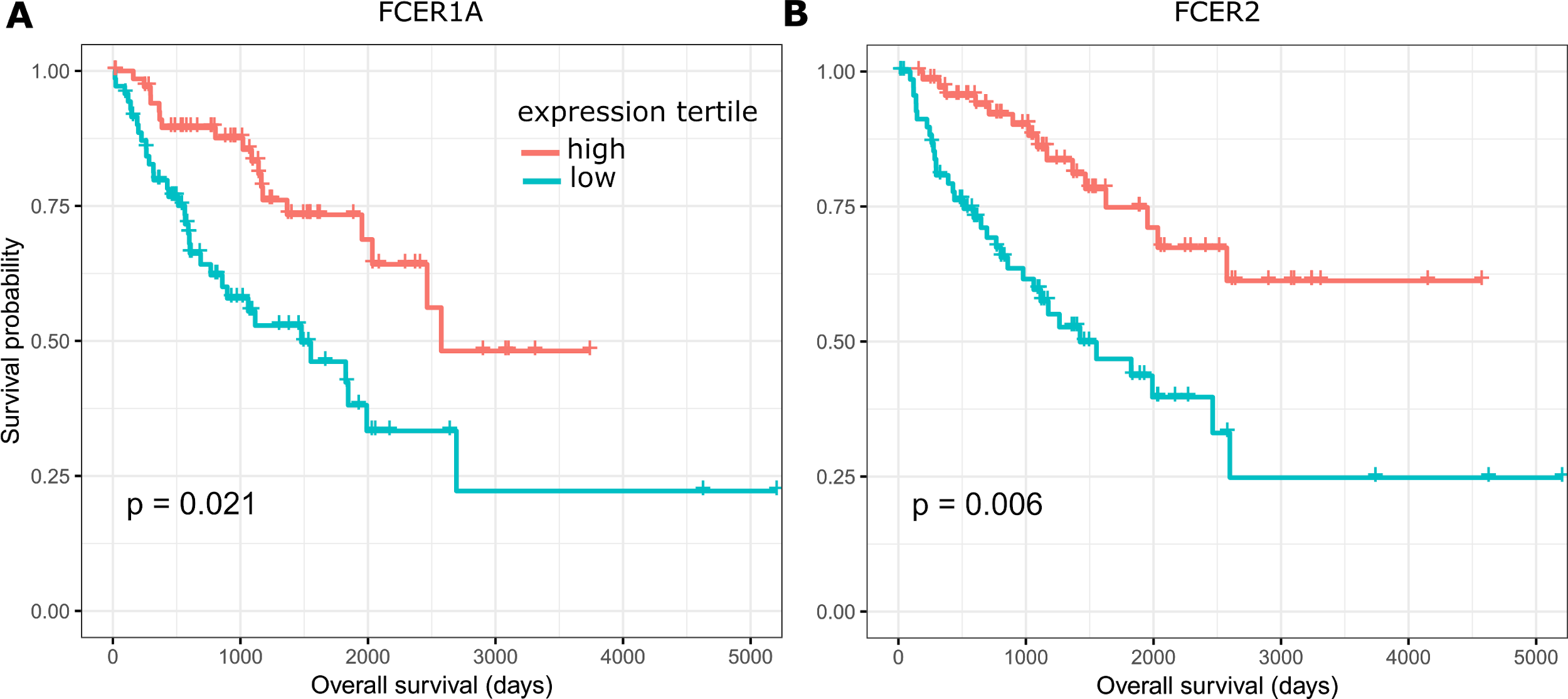


**Additional Figure S9. FCER1A and FCER2 expression as predictor of overall survival.** The overall survival of patients in the upper expression tertile was compared against patients in the lower tertile for the TCGA-SARC cohort using **A** FCER1A expression or **B** FCER2 expression as predictor. P values indicate the results from the log-rank test.


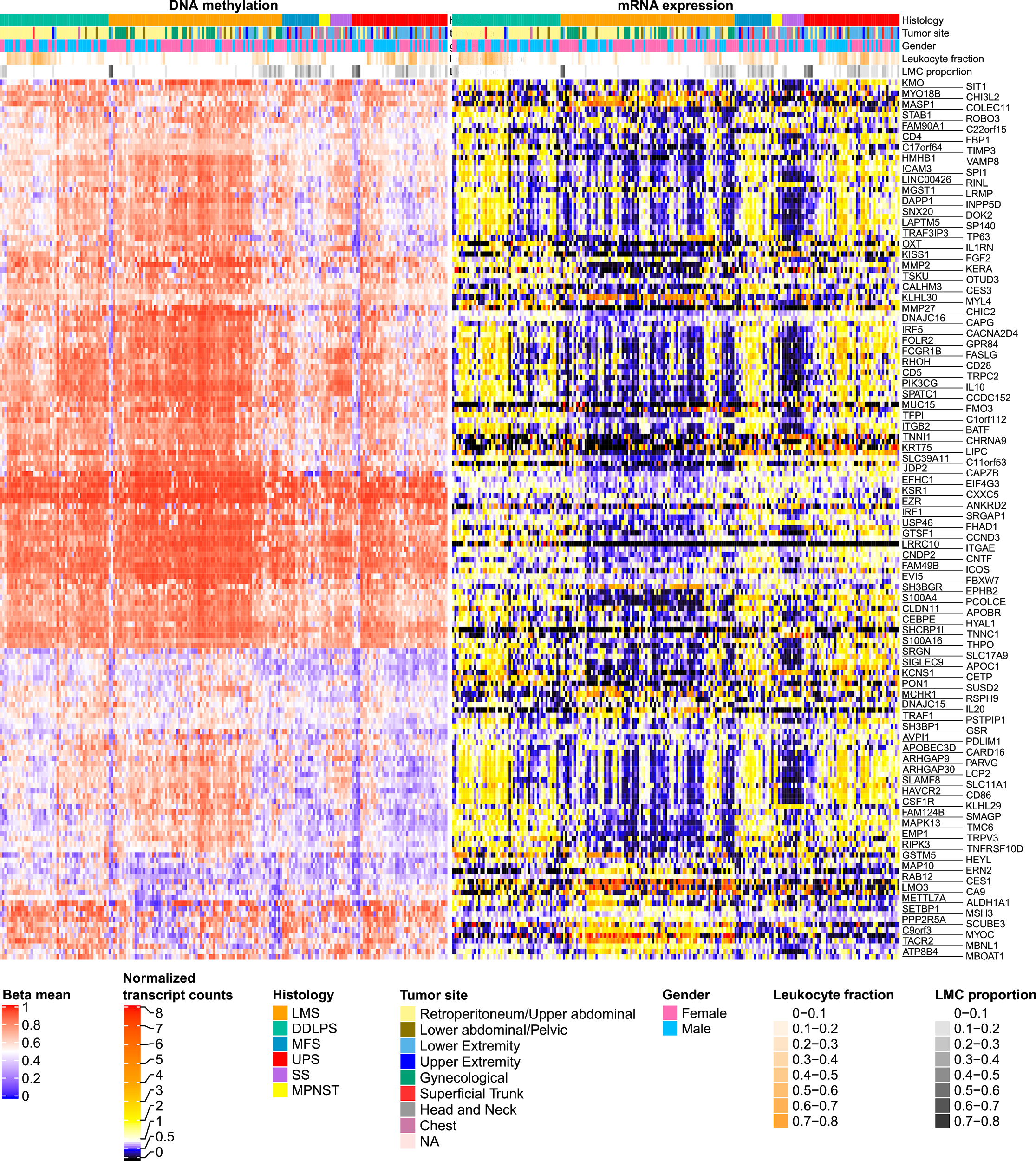


**Additional Figure S10. LMC6 gene signature associated with tumor-infiltrating leukocytes.** The heatmaps show the DNA methylation and corresponding mRNA expression for upregulated genes in samples with a high proportion of LMC6. Samples are clustered within the same histological subtype in columns, and genes are clustered in rows. In contrast to LMC3, the LMC6 proportion has a low correlation with the predicted leukocyte fraction. However, LMC6 gene expression is highly correlated with the leukocyte fraction score.


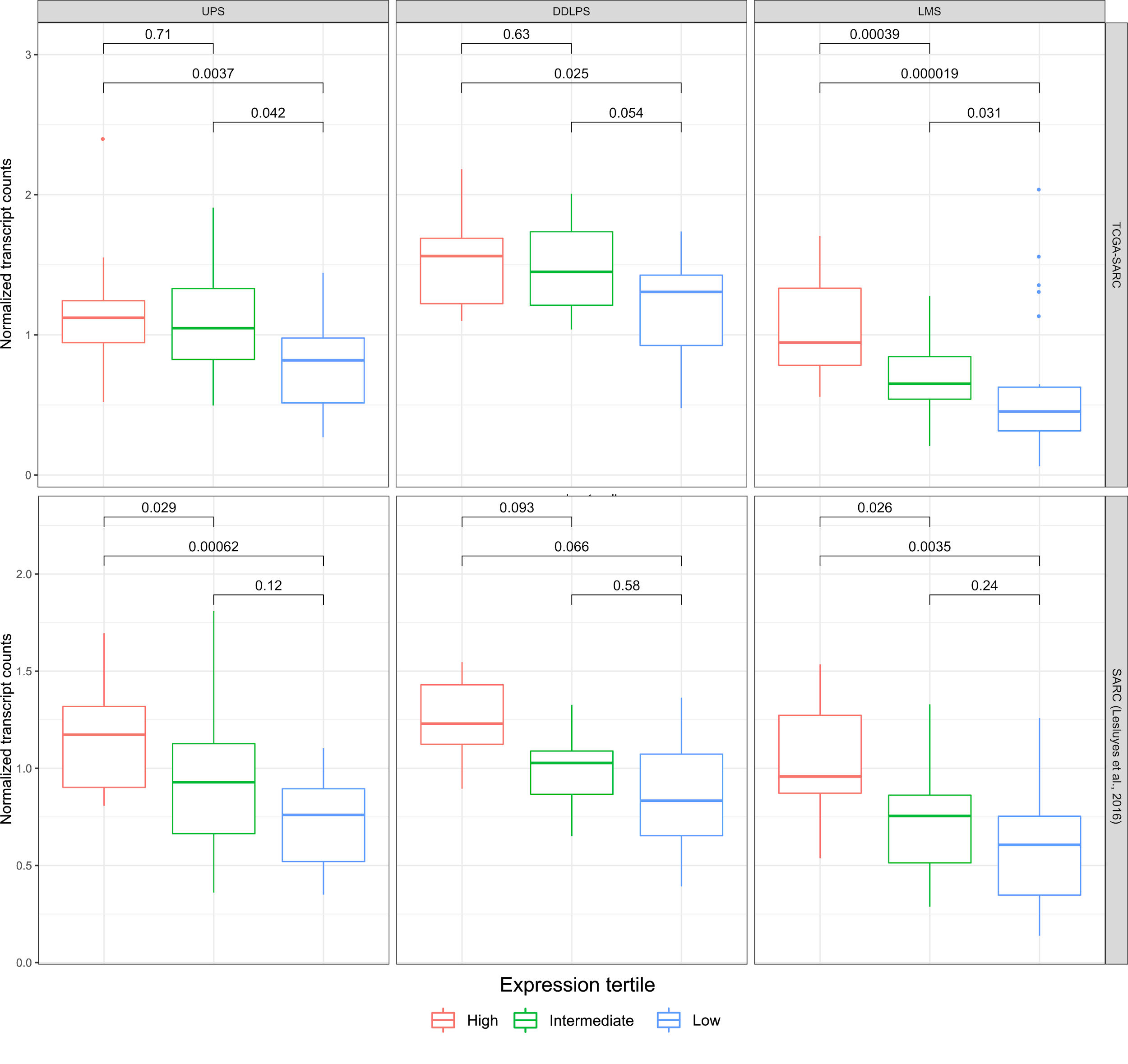


**Additional Figure S11. RB1 expression is depleted in sarcomas with low TIL score.** UPS, DDLPS and LMS samples were grouped into tertiles by their TIL score. The boxplots show the normalized RB1 mRNA expression for each of these subtypes in TCGA-SARC and Lesluyes et al. (2016) cohorts. Pairwise comparisons were performed using the Wilcoxon test.
